# The Power of Profanity: The Meaning and Impact of Swear Words in Word of Mouth

**DOI:** 10.1177/00222437221078606

**Published:** 2022-04-28

**Authors:** Katherine C. Lafreniere, Sarah G. Moore, Robert J. Fisher

**Keywords:** profanity, word of mouth, review, meaning, linguistics

## Abstract

Swearing can violate norms and thereby offend consumers. Yet the prevalence of swear word use suggests that an offensiveness perspective may not fully capture their impact in marketing. This article adopts a linguistic perspective to develop and test a model of how, why, and when swear word use affects consumers in online word of mouth. In two field data sets and four experiments, the authors show that relative to reviews with no swear words, or with non-swear-word synonyms (e.g., super), reviews with swear words (e.g., damn) impact review readers. First, reviews with swear words are rated as more helpful. Second, when a swear word qualifies a desirable [undesirable] product attribute, readers’ attitudes toward the product increase [decrease] (e.g., “This dishwasher is *damn* quiet [loud]!”). Swear words impact readers because they convey meaning about (1) the reviewer and (2) the topic (product) under discussion. These two meanings function as independent, parallel mediators that drive the observed effects. Further, these effects are moderated by swear word number and style: they do not emerge when a review contains many swear words and are stronger for uncensored and euphemistic swear words (e.g., darn) than censored swear words (e.g., d*mn). Overall, swear words in reviews provide value to readers—and review platforms—because they efficiently and effectively convey two meanings.

## Warning

*This article contains strong language that some readers may consider offensive*.

People hear and use swear words more often than ever before (Stapleton 2010): .5% to .7% of all the words spoken in daily conversation are swear words (Jay 2009). This percentage is considerable given that first-person plural pronouns such as “we” and “our”—a central part of speech—occur at a 1% rate (Mehl and Pennebaker 2003). Swearing is even more prevalent online: 7.7% of Twitter posts (Wang et al. 2014) and 8.9% of Yelp reviews (see field data) contain at least one swear word. Despite the frequent use of swear words by consumers, little research in marketing has explored their impact (e.g., Brown and Schau 2001; Dahl, Frankenberger, and Manchanda 2003; Hair and Ozcan 2018).

This limited amount of research is perhaps unsurprising, given the common view that swear words are antisocial and offensive (Rassin and Muris 2005; Robbins et al. 2011; Stapleton 2010). Indeed, the denotative (i.e., literal) meanings of swear words are related to taboo topics (e.g., sex), and swear words are often defined as taboo or offensive words (Jay 2009). Building on this, prior research in marketing has relied on the notion that swear word use violates norms (Brown and Schau 2001). Yet such norm violations can have mixed effects: while swear word use in advertising can increase attention and recall (Dahl, Frankenberger, and Manchanda 2003), swear word use in online reviews can decrease perceptions of the reviewer's objectivity, limiting their effectiveness to only highly positive reviews (Hair and Ozcan 2018). Consistent with this perspective, many websites (e.g., Amazon, Trip Advisor) prohibit the use of swear words.

Despite their taboo origins and definition, research in linguistics suggests that an offensiveness perspective does not provide a complete picture of the impact of swear words. First, under certain conditions, swear words are not considered offensive (Kapoor 2014; Seizer 2011). For example, swear words can express politeness and solidarity when used by factory team members (Daly et al. 2004). Second, swear words have gone through a delexicalization process, such that their original (taboo) meanings have been lost gradually over time (Fairman 2007; Jay 1992). These changes in offensiveness and meaning coincide with the frequent and increasing use of swear words (Jay 2009), which suggests that they are useful to communicators. Yet, beyond their potential to offend, it remains unclear what specific meaning(s) swear words communicate (Jay and Jay 2015).

The current research builds on and extends work in linguistics to develop a model of how, why, and when swear words affect consumers in the context of online word of mouth (WOM; i.e., reviews). Specifically, we argue that swear words function as mixed-meaning expressions, which are defined as words that convey two meanings (Gutzmann and Turgay 2012). We propose that swear words in reviews convey meaning about (1) the reviewer's feelings and (2) the product under discussion. We hypothesize that these two meanings function as independent, parallel mediators, such that swear words convey both meanings, which both impact review readers. For example, consider a swear word used as a degree adverb: “This TV show is *damn* boring.” This swear word should communicate that both (1) the reviewer's feelings about the show and (2) the show's attribute of “boring” hold to a high degree. Accordingly, swear words in reviews may be helpful to readers and may positively (e.g., the show is *damn* funny) or negatively (e.g., the show is *damn* boring) impact outcomes such as readers’ product attitudes. Further, we identify moderators that attenuate the effect of swear words on readers (e.g., the number of swear words in a review). We test our model across two field data sets and four experiments.

This research develops and tests a comprehensive, linguistics-based model of swear word use in WOM, differentiating it from socially based perspectives such as norm violations (Brown and Schau 2001; Dahl, Frankenberger, and Manchanda 2003) and offensiveness (Hair and Ozcan 2018). This linguistic lens enables us to make several contributions to marketing and linguistics. First, we build on and extend previous theorizing in linguistics (Löbner 2013) and provide the first empirical test of the meaning and impact of swear words. Second, we identify two new pathways through which swear words exert their effects on readers. Further, we theorize and show that swear words’ dual meanings function as parallel, equal mediators: a single word can efficiently communicate meaning about the reviewer and the product. Thus, academics and practitioners should consider both inferences in models of WOM. Third, we identify two novel variables that moderate the impact of swearing on readers: swear word number and style. This leads to an important implication both theoretically and practically: factors that change the meanings conveyed by swear words can diminish their value in WOM. Fourth, this research qualifies prior work suggesting that swear words are useful only in extremely positive reviews (Hair and Ozcan 2018). We confirm that swear words are most effective in highly positive reviews but show that swear words are useful to readers regardless of review valence.

Finally, this work offers insight to platforms about how to manage consumer swearing, and highlights when swearing is most valuable. Our findings suggest that platforms may benefit from tolerating, rather than censoring or removing, swear words: swear words are powerful communication tools that help readers, especially when they are uncensored and used sparingly.

## Conceptual Development

### Swear Words and Meaning

Meaning refers to the idea or thing that one intends to communicate (Collins Dictionary 2021). Language philosophers posit that everything that is uttered in an ongoing conversation is relevant (Grice 1975; Searle 1976) and that utterances and symbols are made with specific intentions—they are not merely natural phenomena that do not require deciphering (Searle 1965). Stated differently, when a consumer writes a review using particular words—such as swear words—readers assume they did so for a reason and make inferences about their word use.

A lay theory is that swear words are used only by speakers who lack vocabulary (Pinker 1994) and that they do not constitute genuine language (Jay and Jay 2015). However, linguists argue that swear words ought to convey meaning because they obey syntactic and semantic rules (Dewaele 2015; Jay 2000; Jay and Jay 2013), and because individuals know the rules that govern how and when to swear (Allan and Burridge 2006; Jay 2000; Jay and Jay 2013). Yet, beyond their original denotative meaning, it remains unclear what meaning swear words might communicate (Jay and Jay 2015), and with what effects. We build on recent work in linguistics to argue that swear words in reviews function as mixed-meaning expressions. Mixed-meaning expressions are words that convey meaning about (1) the speaker and (2) the topic under discussion (i.e., the product being reviewed; Gutzmann and Turgay 2012).

#### Speaker meaning

First, linguists have theorized that swear words convey meaning about the speaker's feelings (i.e., expressive meaning or language intensity; e.g., Blakemore 2011; Hobbs 2013; Löbner 2013). Specifically, swear words convey the strength of the speaker's feelings about some state of affairs (Löbner 2013)—they increase the emotional potency of a particular sentence or expression to a high degree. The inference of strong feelings arises from listeners’ knowledge about swear words’ taboo status (Allan and Burridge 2006; Stapleton 2010). Indeed, people often use swear words when they express strong attitudes or emotions (e.g., “*Holy shit*, that was fun!”; Jay 2000). Therefore, listeners infer that the speaker has strong feelings because they were willing to take a social risk by using a swear word (Foolen 2015; Jay 2000). Even if the listener is not personally offended by swearing, the act of swearing still conveys meaning about the strength of the speaker's feelings because it is taboo (Hobbs 2013).

Building on this literature, in the context of reviews, we hypothesize that when a reviewer uses a swear word in a particular sentence or expression (e.g., “This TV show is *damn* boring/funny.”), readers should infer that the reviewer has stronger feelings about the topic under discussion (e.g., the show's boringness/funniness) than if they had not used a swear word. In turn, these inferences about the reviewer's feeling strength should affect readers’ attitudes toward the product, because people update their judgments about a given object based on their inferences about others’ feelings toward that object (Schwarz and Clore 1983; Van Kleef 2010).

#### Topic meaning

Second, Löbner (2013) has theorized that swear words in the form of nouns might function as mixed-meaning expressions (see also Blakemore 2015), such that they might convey meaning not only about the speaker's feelings but also about the topic under discussion (i.e., descriptive or propositional meaning; Gutzmann and Turgay 2012). Specifically, Löbner (2013) suggested that when swear words are used as nouns, they identify and describe the subject (e.g., “This *asshole* spilled their drink on me.”).

Building on this, we argue that even when swear words take on forms other than nouns, they can convey meaning about the topic under discussion (i.e., the product). This is because the meaning of an utterance is not solely contingent on its literal meaning (Searle 1976); words can be modified grammatically to convey different meanings (Foolen 2015). Consider, for example, the various ways in which one can use the word “fuck” (Pinker 2007). When the word is used descriptively (e.g., “Let's *fuck*”) or abusively (e.g., “*Fuck* off!”), it serves as a verb and conveys what action the speaker wants the listener to take (even if the action is not based on the word's original denotative meaning). When the word is used idiomatically (e.g., “That's *fucked* up.”), it serves as an adjective and conveys that the speaker thinks the situation is unfortunate and weird. Finally, when the word is used emphatically (e.g., “This is *fucking* amazing!”) or cathartically (e.g., “*Fuck*! That hurt.”), it serves as an adverb and conveys that the quantity of the word it modifies (e.g., amazingness, pain) holds to a high degree.

In each of these cases, the swear word conveys meaning not only about the speaker (i.e., their strong feelings), but also about the topic under discussion. Stated differently, the swear word functions syntactically like a content word and provides information about what is being discussed. The specific meaning conveyed about the topic under discussion depends on how the swear word is modified grammatically. For example, swear words used as nouns describe the subject (Löbner 2013), whereas swear words used as adjectives or adverbs describe or quantify the subject's attributes. In the context of reviews, regardless of a swear word's specific form, we propose that readers will use the meaning it conveys to make inferences about the product under review; in turn, this should impact their attitudes (He and Bond 2013; Moore 2015).

#### Parallel mediation

**Figure 1.**
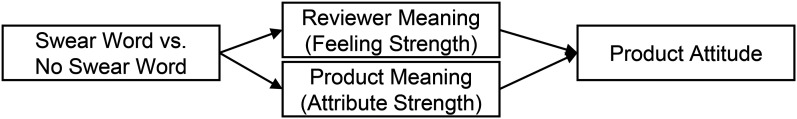
The meanings conveyed by swear words in reviews.

Thus far, we have argued that swear words convey meaning about (1) the speaker and (2) the topic under discussion. We suggest that these meanings function as independent, parallel mediators because they are drawn about different objects (the reviewer and the product), which have each been shown to affect readers in prior work (e.g., the reviewer in Hamilton, Vohs, and McGill [2014] and Hair and Ozcan [2018], the product in Kivetz and Simonson [2000]). We affirm the independence of these two meanings empirically and test our proposed parallel process via mediation and moderation. Overall, swear words in reviews should lead to inferences about the reviewer and the product, which should both affect readers’ product attitudes (Figure 1; e.g., Hamilton, Vohs, and McGill 2014; He and Bond 2013).

### Swear Words as Degree Adverbs

While we consider swear words in all grammatical forms in the field data, our experiments focus on swear words when they are used emphatically (e.g., “*Fucking* awesome!”) and cathartically (e.g., “*Holy shit*!”). This is because speakers most often use swear words in these grammatical forms (Jay 1992), and because these usages do not reflect the swear word's original meaning and therefore cannot be interpreted literally. Critically, these forms constrain the product inferences that can be drawn from the use of a swear word, which enables us to compare swear words to non-swear-word alternatives. Such a comparison will allow us to assess whether swear words in reviews are uniquely valuable or replaceable.

When used emphatically or cathartically, swear words function as degree adverbs. Degree adverbs quantify the word they modify (e.g., “the dishwasher is *extremely* quiet!”) and communicate that the word being modified holds to a high degree (e.g., level of quietness; Gutzmann and Turgay 2012; Van der Wouden and Foolen 2017). In the context of reviews, swear words used as degree adverbs should lead to inferences about the level to which a product holds a particular attribute (e.g., quietness). For simplicity, we refer to swear words used as degree adverbs as “degree swear words” (e.g., “the dishwasher is *fucking* quiet”).

Following our theorizing, compared with a non-swear-word synonym (e.g., “extremely”), we expect a degree swear word to convey stronger reviewer feelings and stronger product attributes: the reviewer has stronger feelings about the dishwasher's quietness, and the dishwasher has a higher degree of quietness. Degree swear words should convey a higher level of the attribute they qualify because swear words are negative words (Van der Wouden and Foolen 2017). As in other areas of judgment and cognition (Baumeister et al. 2001), there is a negativity bias in language processing: negative words draw more attention than neutral or positive words (Foolen 2015; Jing-Schmidt 2007). Thus, when negative words are used as degree adverbs, they should lead to a higher degree of intensification than neutral or positive words because they build on the literal (negative) meaning of the word (Foolen 2015). As theorized previously, using a degree swear word (vs. non-swear-word synonym) should also convey stronger reviewer feelings because the act of swearing breaks a taboo (Allan and Burridge 2006; Stapleton 2010).

We can also compare degree swear words with negative words that have been modified grammatically into degree adverbs. For example, the word “insane” can be modified from an adjective into a degree adverb: “The dishwasher is *insanely* quiet.” While degree swear words and negative words should convey similarly high levels of the product's attribute (i.e., quietness), using a degree swear word (vs. negative word) should convey stronger reviewer feelings because it breaks a taboo (Allan and Burridge 2006; Stapleton 2010).

**Figure 2.**
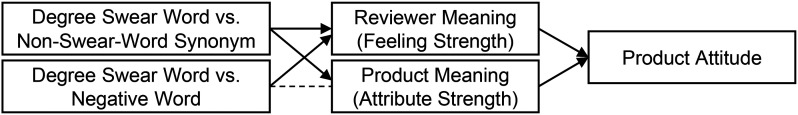
Swear words as degree adverbs in reviews.

Overall, degree swear words (vs. non-swear-word synonyms or negative words) should have the greatest impact on readers because of these differences in meaning (Figure 2). A degree swear word quantifying a desirable attribute (e.g., “The dishwasher is *damn* quiet.”) should enhance readers’ attitudes toward the product, while a swear word quantifying an undesirable attribute (e.g., “The dishwasher is *damn* loud.”) should decrease readers’ attitudes.

### The Diagnosticity of Swear Word Meaning

We posit that the effect of swear words on readers’ product attitudes will be moderated by factors that change the meanings they convey. Thus, swear words should be used to make inferences only when they are diagnostic: when they help readers distinguish between alternative hypotheses, interpretations, or categorizations, such as whether a product is high or low quality (Feldman and Lynch 1988; Herr, Kardes, and Kim 1991). We consider two factors that change a swear word's meaning: swear word number and swear word style.

#### Swear word number

**Figure 3.**
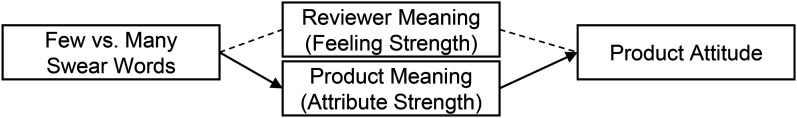
Using a few (vs. many) swear words in reviews.

We expect swear words to be diagnostic for inferences about the product depending on the number of swear words a review contains. Here, we rely on the notion that readers assess their potential product satisfaction by determining whether the product outcome in a review can be attributed to the product (e.g., quality) or the reviewer (e.g., personal taste; Chen and Lurie 2013; He and Bond 2015). When relatively few degree swear words are used, they should be diagnostic of the product's attribute: their limited use conveys discriminatory information about the strength of the attribute. However, when many degree swear words are used, they should be less diagnostic of the product's attribute: their excessive use conveys nondiscriminatory information about attribute strength, because it becomes unclear if the reviewer is swearing to convey the degree of the product's attribute or for other, dispositional reasons (e.g., they are exaggerating or are prone to strong feelings). Thus, relative to using few swear words, using many swear words should convey *lower* attribute strength, resulting in weaker effects on readers’ product attitudes. However, because the act of swearing itself breaks a taboo (Allan and Burridge 2006; Stapleton 2010), using any number of swear words (few or many) should convey similar levels of the reviewer's feelings (Figure 3).

#### Swear word style

Swear words may be presented in different speech styles: uncensored (e.g., *fuck*), euphemistic (e.g., *frick*), and censored (e.g., f***; Allan and Burridge 2006). First, we expect uncensored and euphemistic swear words to convey similar product and reviewer meanings. Euphemistic swear words are indirect expressions of taboo topics. A lay theory is that euphemistic swear words are polite and therefore different from uncensored swear words (Burridge 2012). However, swear words are taboo because of what they represent, not because of the words themselves. Thus, euphemistic swear words are still meaningful because they maintain their association with taboo topics (Burridge 2012). Further, euphemistic swear words are pronounced similarly to uncensored swear words, which helps maintain their negative connotations. Phonetically similar words are connected because of the strong link between sound and sense (Allan and Burridge 2006). Swear words are so potent that even innocent vocabulary words are affected through spurious association. For example, the Canadian city Regina makes some people uncomfortable because of its phonetic similarity to “vagina” (Allan and Burridge 2006). Thus, uncensored and euphemistic swear words should have a similar impact on readers’ product attitudes because they convey the same meanings about the product and the reviewer.

**Figure 4.**
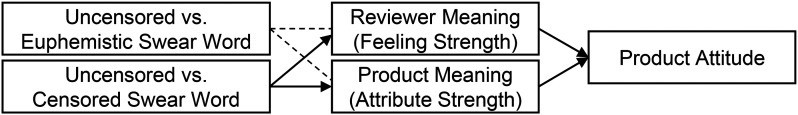
Censored, euphemistic, and uncensored degree swear words in reviews.

Second, we expect an uncensored (vs. censored) swear word to convey stronger product attitudes and stronger reviewer feelings. Censored swear words use symbolic stand-ins or acronyms to replace swear words. This modification not only reduces the phonetic link to uncensored swear words but also enables speakers to openly suppress the obscenity (Allan and Burridge 2006). Thus, censored swear words should be less meaningful because they provide less information about the topic (the product), and it becomes less clear if the reviewer feels strongly. Overall, an uncensored (vs. censored) swear word should have greater effects on readers’ product attitudes (Figure 4).

## Overview of Studies

We test these predictions across two field data sets and four experiments. First, we analyze field data from Yelp and Amazon to examine the impact of swear words (vs. no swear words) on review helpfulness ratings. We test our two moderating variables by examining the effects of swear word number and by comparing the use of different swear word styles (uncensored, euphemistic, and censored) to no swear words. Finally, we affirm that the effects of swear words persist across review valence (positive, neutral, and negative star ratings). Second, we report four experiments that provide causal evidence via mediation and moderation for the observed effects, test our proposed parallel mediation model, and reexamine our moderators. Experiments 1a, 1b, and 2 provide process evidence by measuring the two meanings drawn from swear words (Figure 1). Experiment 1b offers a stringent test of our model by comparing swear words to non-swear-word synonyms (Figure 2). Experiments 2 and 3 test swear word number (Figure 3) and swear word style (Figure 4) as moderators, respectively.

## Field Data

To explore the idea that swear words can be useful to readers, we obtained field data from two leading review websites, Yelp and Amazon. We use these data to test whether swear word use impacts review helpfulness ratings. These ratings reflect readers’ judgments that a review can reduce uncertainty, guide decision making, and influence purchase decisions (Chen, Dhanasobhon, and Smith 2008; Moore 2015; Zhu, Yin, and He 2014). We predict that reviews containing swear words (vs. no swear words) will be more helpful to readers.

This study serves two additional purposes. First, we test both moderators, swear word number and style. For number, we predict that a few swear words (vs. no swear words) should increase review helpfulness but many swear word (vs. no swear word) should not. For style, we categorized swear words as uncensored (e.g., “fuck”), euphemistic (e.g., “frick”), or censored (e.g., “f***”); we predict that uncensored or euphemistic swear words (vs. no swear words) should increase review helpfulness but censored swear words (vs. no swear words) should not. Second, we test the effect of swear words across review valence. Consistent with our conceptual framework (Figure 1), swear words should convey meaning and provide value regardless of valence. Relative to no swear words, swear words in negative reviews should lead to negative inferences (e.g., “This dishwasher is damn loud!”) and increase review helpfulness. Similarly, swear words in positive reviews should lead to positive inferences and increase review helpfulness. Consistent with prior work (Hair and Ozcan 2018), swear words may be the most helpful in positive reviews.

### Method

Yelp reviews were obtained from the 2017 Yelp Dataset Challenge. This publicly available data set contained all reviews as of January 20, 2017, that cleared Yelp's software, which automatically screens out fake or untrustworthy reviews. The data set consisted of approximately 4.7 million reviews of 156,000 businesses in 12 metropolitan areas from four countries (Yelp 2017). One hundred thousand of these reviews were randomly selected for analysis. In this final data set, there were 76,544 unique reviewers for 42,883 different businesses. For each review, the data included review text, star rating (a five-point rating system, with five stars being the best), date posted, and number of people who voted the review as useful.

Amazon reviews were obtained from a publicly available repository (He and McAuley 2016). The data set contains 82.8 million product reviews from May 1996 to July 2014. Two hundred thousand of these reviews were randomly selected for analysis. In the final data set, there were 190,240 unique reviewers for 161,092 different products across 24 product categories (e.g., books, baby, electronics; see Web Appendix A). For each review, the data included review text, star rating (a five-point rating system, with five stars being the best), date posted, number of people who voted the review as helpful, and number of people who voted the review as unhelpful.

Some key differences between these data sets make their analyses distinct yet complementary. First, Yelp's guidelines state that swear words are allowed in reviews, whereas Amazon's guidelines state that reviews containing swear words are not allowed and may be removed or rejected. Second, Yelp allows readers to vote a review only as useful, resulting in a count dependent variable that does not reveal how many readers found the review not useful. Alternatively, Amazon allows readers to vote a review as helpful *or* unhelpful, resulting in a dependent variable that is the number of helpful votes in proportion to the total number of votes. Third, Yelp reviews are written primarily about services (e.g., restaurants, excursions, repairs), whereas Amazon reviews are written primarily about products (e.g., books, clothes, pet supplies). Overall, analyzing both data sets enable a robust examination of the swearing effect.

### Independent Measure

Using Linguistic Inquiry and Word Count (LIWC) software (Pennebaker et al. 2015), we identified all the reviews that contained at least one swear word. LIWC categorizes words into validated, preexisting dictionaries (Tausczik and Pennebaker 2010) and reports the proportion of words in a text that fall into each dictionary (e.g., positive emotion). We updated LIWC's swear word dictionary of 53 word stems so that it (1) excluded words that did not function as swear words in the review context (e.g., the word “bloody” was excluded because it was used in the Yelp data set primarily in reference to a Bloody Mary cocktail) and (2) included some swear words not in the dictionary (e.g., frick, f*ck). The final updated dictionary contained 145 word stems and allowed us to analyze uncensored, censored, and euphemistic swear words in one all-inclusive swear word variable, as well as to analyze each swear word style as a separate variable (for coding details, see Web Appendix B). These binary variables were set to 1 when a review contained one or more swear words and 0 otherwise. Of the 100,000 Yelp reviews, 8,947 (8.9%) reviews contained a swear word. Of the 200,000 Amazon reviews, 6,965 (3.5%) reviews contained swear words.

#### Dependent measure

Following prior research, the dependent measure for a Yelp review was operationalized as the number of “useful” votes it received (M = 1.01, SD = 2.44; Chen and Lurie 2013), and the dependent measure for an Amazon review was the number of helpful votes received in proportion to the total number of votes received (M = 32.82%, SD = 43.59%; Chen, Dhanasobhon, and Smith 2008).

#### Control variables

We controlled for other review characteristics that could affect review helpfulness: the number of months between review posting and data extraction (Yelp: January 21, 2017, M = 32.01, SD = 25.66; Amazon: July 24, 2014, M = 28.59, SD = 35.56; Zhu, Yin, and He 2014), review valence via the five-point star rating (Yelp: M = 3.73, SD = 1.40; Amazon: M = 4.17, SD = 1.25; Chen and Lurie 2013; Zhu, Yin, and He 2014), and the number of words in the review (Yelp: M = 117.64, SD = 109.77; Amazon: M = 92.01, SD = 123.97; Zhu, Yin, and He 2014). We note, however, that the effect of review length on helpfulness is curvilinear (an inverted U-shape) because particularly long reviews are difficult to absorb and therefore negatively affect value (Schindler and Bickart 2012). Indeed, the quadratic regression line for review length significantly improved its prediction of the dependent variable relative to the linear regression line for both data sets (Yelp: R^2^ change = .003, F(1, 99,997) = 355.86, *p* < .001; Amazon: R^2^ change = .014, F(1, 199,997) = 3,081.12, *p* < .001). Thus, we applied a square transformation to the review length variable (Yelp: M = 25,866.82, SD = 62,733.06; Amazon: M = 23,833.80, SD = 160,365.87).

## Results

We used negative binomial regression because the dependent variable value is a count variable and its variance exceeds its mean (Yelp: M_useful votes_ = 1.01, Var = 5.94; Amazon: M_proportion helpful votes_ = 32.82%, Var = 1,899.74%), and because the dispersion coefficients in both data sets were positive and significant (Yelp: B = 1.76; 95% confidence interval [CI] = [1.73, 1.79]; Amazon: B = 9.83; 95% CI = [9.75, 9.91]), making this model more appropriate than a Poisson model (Greene 2008).

In a base model without control variables, Yelp reviews with swear words received more useful votes than Yelp reviews without swear words (B = .66, Wald χ^2^ (1, n = 100,000) = 1,333.26, *p *< .001; exponentiated B = 1.94). Controlling for variables that could also conceivably affect useful votes (months posted, review valence, and review length) did not change this result (B = .32, Wald χ^2^ (1, n = 100,000) = 316.59, *p *< .001; exponentiated B = 1.37).

Similarly, Amazon reviews with swear words received a higher proportion of helpful votes than Amazon reviews without swear words (B = .33, Wald χ^2^ (1, n = 200,000) = 71.78, *p *< .001, exponentiated B = 1.40), and this result did not change when the control variables were added to the model (B = .16, Wald χ^2^ (1, n = 200,000) = 16.02, *p *< .001, exponentiated B = 1.17). Additional robustness checks using a three-level categorical variable for star ratings and using LIWC's original swear word dictionary supported these results (Web Appendices C and D).

### Swear word number

We predicted that few swear words (vs. no swear words) in reviews would increase review helpfulness but many swear words (vs. no swear words) would not. We used the number of swear words as a categorical variable, to compare reviews at each number of swear words (one, two, three, etc.) to reviews containing no swear words (zero; the control condition).

For Yelp reviews, the results of a negative binomial regression (including the control variables) showed that reviews containing one swear word received significantly more useful votes than those with no swear words (n_1 SW_ = 6,940, B = .30, Wald χ^2^ (1, n = 100,000) = 223.05, *p *< .001; exponentiated B = 1.35). Reviews containing two swear words also received significantly more useful votes relative to reviews with no swear words (n_2 SWs_ = 1,363, B = .37, Wald χ^2^ (1, n = 100,000) = 75.87, *p *< .001; exponentiated B = 1.45). Reviews containing three swear words evoked the largest positive effect on useful votes relative to reviews with no swear words (n_3 SWs_ = 381, B = .54, Wald χ^2^ (1, n = 100,000) = 48.68, *p *< .001; exponentiated B = 1.71). The effect became weaker and not significant at four swear words (n_4 SWs_ = 147, B = .18, Wald χ^2^ (1, n = 100,000) = 2.14, *p* = .17; exponentiated B = 1.20) and five or more swear words (n_5+ SWs_ = 116, B = .18, Wald χ^2^ (1, n = 100,000) = 1.56, *p* = .21; exponentiated B = 1.19).

Similarly for Amazon reviews, the results of a negative binomial regression (including control variables) showed that reviews containing one swear word received a higher proportion of helpful votes than those with no swear words (n_1 SW_ = 5,674, B = .16, Wald χ^2^ (1, n = 200,000) = 13.36, *p *< .001). Reviews containing two swear words evoked a larger, but insignificant, effect relative to reviews with no swear words (n_2 SWs_ = 908, B = .16, Wald χ^2^ (1, n = 200,000) = 2.24, *p* = .13). The effect of swear words on the proportion of helpful votes was insignificant at three swear words (n_3 SWs_ = 238, B = .18, Wald χ^2^ (1, n = 200,000) = .81, *p* = .37), four swear words (n_4 SWs_ = 77, B = −.11, Wald χ^2^ (1, n = 200,000) = .09, *p* = .76), and five or more swear words (n_5+ SWs_ = 69, B = .21, Wald χ^2^ (1, n = 200,000) = .36, *p* = .55; Table 1).

**Table 1.** The Effect of Swear Word Number.

Variables	Yelp (Useful Votes)		Amazon (Proportion of Helpful Votes)	
	B (SE)	Exp(B)	B (SE)	Exp(B)
Intercept	−.23*** (.02)	.795	3.18*** (.03)	23.92
5 + swear words	.18 (.14)	1.19	.23 (.38)	1.25
4 swear words	.18 (.13)	1.20	−.11 (.36)	.90
3 swear words	.54*** (.08)	1.71	.18 (.20)	1.20
2 swear words	.37*** (.04)	1.45	.16 (.11)	1.17
1 swear word	.30*** (.02)	1.35	.16*** (.04)	1.17
Months posted	.01*** (.0002)	1.01	.01*** (.0002)	1.01
Review valence	−.08*** (.004)	.92	−.03*** (.01)	.98
Review length^a^	.00001*** (.0000001)	1.00	.000001*** (.0000001)	1.00
Dispersion	1.76* (.02)		9.83* (.04)	
Pearson χ^2^	193,088.70		44,642.38	

*p ≤ .05.

**p ≤ .01.

***p ≤ .001.

^a^ Square transformed,

### Swear word style

We predicted that uncensored or euphemistic swear words (vs. no swear word) would positively impact review helpfulness but censored swear words (vs. no swear words) would not. We modeled the three styles of swear words as separate independent variables (see Tables 2 and 3 for the distribution; 0 = absent; 1 = present).

**Table 2.** Descriptive Statistics.

Variable	Yelp's Useful Votes		Amazon's Proportion of Helpful Votes	
	Present Mean (SD)	Absent Mean (SD)	Present Mean (SD)	Absent Mean (SD)
All swear words	1.81 (4.00)	.94 (2.21)	44.97 (43.56)	32.38 (32.38)
Uncensored	1.82 (4.13)	.95 (2.24)	45.03 (43.45)	32.47 (43.54)
Euphemistic	2.05 (5.36)	1.00 (2.37)	46.90 (44.49)	32.73 (43.57)
Censored	1.97 (3.74)	1.01 (2.42)	43.09 (42.48)	32.79 (43.59)

**Table 3.** The Effect of Swear Word Style.

Variables	Yelp (Useful Votes)		Amazon (Proportion of Helpful Votes)	
	B (SE)	Exp(B)	B (SE)	Exp(B)
Intercept	−.22*** (.02)	.80	3.18*** (.03)	23.93
Uncensored	.29*** (.02)	1.34	.13** (.04)	1.14
Euphemistic	.37*** (.04)	1.44	.19* (.09)	1.21
Censored	.10* (.05)	1.00	.12 (.12)	1.13
Months posted	.01*** (.0002)	1.01	.01*** (.0002)	1.01
Review valence	−.08*** (.004)	.92	−.03*** (.01)	.98
Review length^a^	.00001*** (.0000001)	1.00	.000001*** (.0000001)	1.00
Dispersion	1.76* (.02)		9.83* (.041)	
Pearson χ^2^	190,270.30		44,638.09	

*p ≤ .05.

**p ≤ .01.

***p ≤ .001.

^a^ Square transformed.

For Yelp reviews, the results of a negative binomial regression (controlling for months posted, review valence, and review length) showed that reviews received more useful votes when uncensored swear words were present (vs. absent; n = 7,331, B = .29, Wald χ^2^ (1, n = 100,000) = 226.67, *p *< .001), when euphemistic swear words were present (vs. absent; n = 1,429, B = .37, Wald χ^2^ (1, n = 100,000) = 77.47, *p *< .001), and, surprisingly, when censored swear words were present (vs. absent; n = 889, B = .10, Wald χ^2^ (1, n = 100,000) = 3.74, *p* = .05).

For Amazon reviews, the results of a negative binomial regression (including control variables) showed that reviews received a higher proportion of helpful votes when uncensored swear words were present (vs. absent; n = 5,490, B = .13, Wald χ^2^ (1, n = 200,000) = 9.32, *p* = .002) and when euphemistic swear words were present (vs. absent; n = 1,209, B = .1.92, Wald χ^2^ (1, n = 200,000) = 4.48, *p* = .034). However, as we expected, there was no significant difference in the proportion of helpful votes for reviews when censored swear words were present versus absent (n = 611, B = .12, Wald χ^2^ (1, n = 200,000) = .85, *p* = .35).

### Review valence

We predicted that swear words would be useful in both negative and positive reviews. To test the effect of swear words across review valence, we split the data sets into negative (one or two stars), neutral (three stars), and positive (four or five stars) reviews and reran the negative binomial regression models (controlling for months posted and review length). The Yelp data showed that reviews containing swear words (vs. no swear words) were more valuable across all review valence categories and were most valuable for positive-valence reviews (B_negative_ = .23, Wald χ^2^ (1, n = 21,583) = 70.20, *p *< .001; B_neutral_ = .25, Wald χ^2^ (1, n = 12,443) = 26.05, *p *< .001; B_positive_ = .38, Wald χ^2^ (1, n = 65,964) = 216.32, *p *< .001). We found similar results when we added an interaction term for the presence of swear words and star ratings to the original model (interaction: B = .11, Wald χ^2^ (1, n = 100,000) = 97.23, *p *< .001).

The Amazon data showed directionally similar effects on the proportion of helpful votes, though it was only significant for positive reviews (B_negative_ = .06, Wald χ^2^ (1, n = 26,053) = .87, *p* = .35; B_neutral_ = .15, Wald χ^2^ (1, n = 17,154) = 1.46, *p* = .23; B_positive_ = .19, Wald χ^2^ (1, n = 156,793) = 13.60, *p *< .001). We found similar, but weaker, results when we added an interaction term for the presence of swear words and star ratings to the original model (interaction: B = .04, Wald χ^2^ (1, n = 200,000) = 3.03, *p* = .08).

### Discussion

An analysis of Yelp and Amazon data showed that the presence (vs. absence) of swear words in reviews increased review helpfulness (Table 4). For swear word number, these results showed that, compared with no swear words, a few swear words increased review helpfulness, but many swear words did not. Specifically, one to three swear words (vs. no swear words) in a review increased review helpfulness. However, this effect was attenuated when the review contained four or more swear words. For swear word style, the results differed somewhat across the data sets. In the Yelp data, the presence (vs. absence) of uncensored, euphemistic, or censored swear words increased the number of useful votes. In the Amazon data, the presence of uncensored and euphemistic swear words also increased review helpfulness, but censored swear words did not. These differences may be due to the platforms' different guidelines about swearing. In addition, consistent with our theorizing, censored swear words may be less valuable to readers because they convey less diagnostic meaning. We address this issue with a formal test of swear word style in Experiment 3.

**Table 4.** Summary of Field Data Results.

Variable Present (vs. Absent)	Yelp	Amazon
Swear words	✓	✓
Uncensored swear words	✓	✓
Euphemistic swear words	✓	✓
Censored swear words	✓	✗
Few swear words (1–3)	✓	✓
Many swear words (4+)	✗	✗

Notes: ✓ = significant difference; ✗ = no difference.

Finally, consistent with Hair and Ozcan's (2018) recent findings, swear words were most helpful in positive reviews. However, consistent with our model, swear words were valuable to readers in both negative and positive reviews. We corroborated this finding in an experiment that manipulates review valence (Web Appendix E). As we expected, relative to no swear words, a swear word in a negative review (e.g., “This dishwasher is damn loud!”) increased review helpfulness and decreased readers’ attitudes toward the product. Similarly, a swear word in a positive review increased review helpfulness and readers’ attitudes toward the product.

Building on the field data results, we conduct a series of experiments to provide a causal test our model. In these experiments, we focus on degree swear words and on positive reviews where swear words qualify positive product attributes. We do so because of the abundance of research suggesting that swear words have negative effects (e.g., Pinker 1994; Rassin and Muris 2005) and because there are far more positive than negative reviews (Woolf 2014).

## Experiment 1a: Swear Word Versus No Swear Word

The purpose of Experiment 1a is to provide a causal demonstration of the field data effect and to test process (Figure 1). We do so by comparing the presence versus absence of a degree swear word in a positive review. We hypothesize that when a swear word qualifies a desirable product attribute, it will convey meaning about the strength of the reviewer's feelings and the strength of the product's attribute, and this will have a positive effect on product attitudes. Further, we expect these two inferences to function as parallel, independent mediators of the effect of swear words on product attitudes.

### Participants, Design, and Measures

We recruited 215 individuals to complete a single-factor (swear word: present vs. absent) between-subjects study (Amazon Mechanical Turk [MTurk]; M_age_ = 35.5 years; 53% male). Twenty-nine participants were excluded for failing an attention check (participants were asked to report how the reviewer described the product's size), leaving a final sample of 186.

Participants were asked to imagine that they wanted to buy a new external battery (i.e., power station) for their electronic devices. Then they were shown a seller's website with an image and a product description of an external battery, along with one positive review that rated the product four out of five stars (Web Appendix F). The title of the review contained the swear word manipulation. In the swear-word-present condition, the title read, “It charged my phone fucking fast.” In the swear-word-absent condition, the title read, “It charged my phone fast.” The remaining text of the review was the same across both conditions: “It's handy and portable. But it feels heavy. It holds a charge fine and its size is okay.”

Participants’ attitude toward the power station was measured with six items using seven-point semantic differential scales, anchored as follows: “negative–positive,” “dislike–like,” “good–bad,” “unfavorable–favorable,” “unappealing–appealing,” and “unpleasant–pleasant” (M = 5.70, SD = .83; α = .91). Attribute strength (i.e., charging speed) was measured with six items. Participants reported the degree to which the power station's charging capabilities were fast-paced, high-speed, turbo, rapid, quick, and swift (1 = “not at all,” and 7 = “very much”; M = 5.56, SD = 1.09; α = .95). Finally, feeling strength was measured with three items by asking participants if they would describe the reviewer's feelings about the product as strong, intense, and confident (1 = “not at all,” and 7 = “very much”; M = 5.28, SD = 1.27; α = .86). As a manipulation check, participants were asked if they found the review to be offensive (1 = “not at all,” and 7 = “very much”; M = 2.20, SD = 1.96) and if they thought most people would find the review to be offensive (1 = “not at all,” and 7 = “very much”; M = 2.78, SD = 2.01).

For brevity, across studies, we report factor analyses on the items measuring feeling strength, attribute strength, and product attitude, as well as manipulation check results for offensiveness, in Web Appendices G and H. For the factor analyses, three factors consistently emerged. Further, as we expected, those in the swear word condition consistently reported greater offensiveness. The reported results hold when controlling for offensiveness.

### Results

A parallel multiple mediation model (model 4; Hayes 2013) revealed significant effects when comparing the presence (coded as 1) versus absence (coded as 0) of a degree swear word.

#### Product attitude

An analysis of variance (ANOVA) showed an expected significant total effect of swear word on attitude toward the product (F(1, 184) = 6.17, *p* = .014, $\eta_{p}^{2}$ = .032), such that participants held more favorable attitudes toward the product when the swear word was present (M_present_ = 5.84, SD = .83) compared with when it was absent (M_absent_ = 5.54, SD = .83).

#### Product strength

An ANOVA on attribute strength (i.e., charging speed) revealed a significant effect of swear word (F(1, 184) = 16.37, *p *< .001, $\eta_{p}^{2}$ = .082): participants thought the battery charged faster when the swear word was present (M_present_ = 5.87, SD = 1.01) versus absent (M_absent_ = 5.56, SD = 1.09).

#### Feeling strength

An ANOVA on feeling strength revealed a significant effect of swear word (F(1, 184) = 87.24, *p *< .001, $\eta_{p}^{2}$ = .322), such that participants thought the reviewer had stronger feelings when the swear word was present (M_present_ = 6.00, SD = .82) compared with when it was absent (M_absent_ = 5.28, SD = 1.27).

#### Mediation analysis

Finally, the parallel multiple mediation analysis tested whether the two meanings explained the effect of swear words on readers’ attitudes. In this case, multiple mediation is useful because it tests the effect of each potential mediator while holding constant the other mediator. This lends more confidence to causal claims because it controls for endogeneity and allows a comparison of the size of the indirect effect for each mediator (Hayes 2013).

The indirect effects of the presence (vs. absence) of a degree swear word via attribute strength (partial B = .17, SE = .06, 95% CI = [.07, .29]) and via feeling strength (partial B = .41, SE = .11, 95% CI = [.19, .63]) were both significant. A pairwise comparison between the two indirect effects was not significant (partial B = .23, SE = .14, 95% CI = [−.03, .51]), suggesting that there was no difference in the strength of the mediators, and that both are equally important to the model. The direct effect of swear words on product attitudes was insignificant when controlling for the mediators (B = −.18, SE = .13, 95% CI = [−.43, .07]).

### Discussion

The results of Experiment 1a replicate and extend the results of the field data. We found that compared with no swear word, the presence of a degree swear word qualifying a desirable product attribute increased review readers’ product attitudes. Further, the presence (vs. absence) of a degree swear word conveyed two meanings, about (1) the strength of the reviewer's feelings and (2) the strength of the product's attributes in terms of charging speed. A factor analysis confirmed the independence of these two meanings, and a process analysis showed that they functioned as parallel mediators that each enhanced readers’ product attitudes.

## Experiment 1b: Swear Word Versus Non-Swear-Word Synonyms

The purpose of Experiment 1b is to provide a more stringent test of our model (Figure 2). To do so, we compare a swear word with two other words that could potentially lead to the same inferences as a swear word: “super” and “insanely.” We selected the word “super” because it is a non-swear-word synonym that has been identified as a mixed-meaning expression. That is, similar to degree swear words, the word “super” communicates meaning about both the speaker's strong feelings and the product's strong attribute (Waksler 2012; see also Foolen 2015; Gutzmann and Turgay 2012). We selected the word “insanely” because it is a negative degree adverb that should quantify the attribute it modifies to the extreme end of the scale (Foolen 2015).

We expect the three conditions to lead to differential inferences about the reviewer and the product and, therefore, to differentially affect readers’ product attitudes. Specifically, because they are both negative words, the swear word and the negative word should generally lead to similar inferences about attribute strength, and to greater inferences about attribute strength than the mixed-meaning word. Further, because it breaks a taboo, the swear word should lead to greater inferences about reviewer feeling strength than both the negative word and the mixed-meaning word. Thus, the swear word should have the greatest positive impact on readers’ product attitudes.

This study also tests two alternative explanations. First, given that swear words observed in isolation can increase arousal (Kensinger and Corkin 2004) and that arousal can affect product preferences (Di Muro and Murray 2012), these effects may be driven by readers’ arousal. Second, given that swear words in advertising can increase attention (Dahl, Frankenberger, and Manchanda 2003), the predicted effects can be driven by differences in attention.

### Participants, Design, and Measures

We recruited 317 participants from MTurk (M_age_ = 36.2 years; 52% male). We excluded 21 participants from analysis for failing an attention check (asking participants to report how the reviewer described the product's wash cycle), leaving a final sample of 296. This study was a between-subjects design with three degree adverb conditions: swear word [“damn”] versus mixed-meaning word [“super”] versus negative word [insanely]. Participants were asked to imagine that they needed to buy a new dishwasher. Then they were shown an image of a dishwasher on a seller's website that included one positive review. The title of the review contained the manipulation and read, “The dishwasher is [damn/insanely/super] quiet!” The remaining review text was the same across conditions (“Cycles work as expected. Layout is okay.”). Consistent with prior work, we measured attention using the elapsed time spent reading the review (M = 18.11, SD = 20.22) because longer viewing times provide participants with more opportunity to attend to and process information (e.g., Moore, Hausknecht, and Thamodaran 1986).

After reading the review, participants reported on product attitudes (M = 5.71, SD = .89; α = .95), attribute strength (i.e., degree of quietness), feeling strength (M = 4.68, SD = 1.41; α = .95), and offensiveness (Web Appendix G). Product attitudes, feeling strength, and offensiveness were measured as in Study 1. For attribute strength, participants reported the degree to which the dishwasher was quiet, silent, inaudible, muted, unobtrusive, suppressed, and faint (1 = “not at all,” and 7 = “very much”; M = 5.24, SD = 1.38, α = .91). For arousal, participants reported how they were feeling (1 = “calm,” and 7 = “excited”; M = 3.17, SD = 1.79).

### Results

A parallel mediation model with a multicategorical independent variable (Model 4; Hayes 2013) revealed significant effects when comparing a swear word with a mixed-meaning word and a swear word with a negative word.

#### Product attitude

An ANOVA revealed a significant total effect of degree adverb condition on product attitudes (F(2, 293) = 8.54, *p *< .001, $\eta_{p}^{2}$ = .055). Participants liked the dishwasher more in the swear word condition (M_damn_ = 6.00, SD = .78), compared with the mixed-meaning word condition (M_super_ = 5.64, SD = .89; B = .35, SE = .12, t(293) = 2.85, *p* = .005) and the negative word condition (M_insanely_ = 5.50, SD = .93; B = .50, SE = .12, t(293) = 4.01, *p *< .001).

#### Attribute strength

An ANOVA revealed a significant effect of degree adverb condition on attribute strength (i.e., degree of quietness; F(2, 293) = 3.37, *p* = .036, $\eta_{p}^{2}$ = .022), such that participants perceived the dishwasher to be quieter in the swear word condition (M_damn_ = 5.49, SD = 1.33) compared with the mixed-meaning word condition (M_super_ = 4.99, SD = 1.43; B = .50, SE = .19, t(293) = 2.60, *p* = .010). As we predicted, there was no difference in attribute strength between the swear word and negative word conditions (M_insanely_ = 5.23, SD = 1.32; B = .27, SE = .19 t(293) = 1.23, *p* = .17).

#### Feeling strength

An ANOVA revealed a significant effect of degree adverb condition on feeling strength (F(2, 293) = 6.62, *p* = .002, $\eta_{p}^{2}$ = .043). Participants perceived the reviewer's feelings to be stronger in the swear word condition (M_damn_ = 5.07, SD = 1.40) compared with the mixed-meaning word condition (M_super_ = 4.38, SD = 1.37; B = .70, SE = .20, t(293) = 3.54, *p *< .001) and the negative word condition (M_insanely_ = 4.58, SD = 1.38; B = 49, SE = .20, t(293) = 2.50, *p* = .013).

#### Mediation

The indirect effects of the swear word (vs. mixed-meaning word) via feeling strength (partial B = .16, SE = .06, 95% CI = [.06, .28]) and attribute strength (partial B = .05, SE = .03, 95% CI = [.001, .11]) were significant. Consistent with Experiment 1a, pairwise comparison between the two indirect effects was not significant (partial B = .05, 95% CI = [−.002, .11]). The direct effect of the swear word on product attitudes was insignificant when controlling for the mediators (B = .17, SE = .12, 95% CI = [−.06, .40]).

The indirect effect of the swear word (vs. negative word) via feeling strength was significant (partial B = .12, SE = .05, 95% CI = [.02, .22]). The indirect effect of the swear word via attribute strength was not significant (partial B = .02, SE = .02, 95% CI = [−.01, .08]), showing that, as predicted, the swear word and the negative word led to similar inferences about quietness. The direct effect of the swear word on product attitudes remained significant when controlling for the mediators (B = .37, SE = .12, 95% CI = [.14, .60]).

#### Alternative explanations

An ANOVA on arousal was not significant (F(2, 290) = .79, *p* = .46, partial η^2^ = .005). Further, an ANOVA on attention, measured using time spent reading the review, was not significant (F(2, 293) = .08, *p* = .92, partial η^2^ = .001).

### Discussion

Experiment 1b tested the impact of swear words in reviews by comparing a degree swear word (i.e., “damn”) with a mixed-meaning degree word (i.e., “super”) and a negative degree word (i.e., “insanely”). The data demonstrate the unique effects of swear words. Even when compared with a mixed-meaning degree word, the degree swear word led to inferences about the reviewer's stronger feelings and the product's stronger attribute of quietness, and these inferences independently and equally enhanced readers’ product attitudes. Further, while both the negative degree word and degree swear word led to similar inferences about the product's quietness, only the degree swear word increased inferences of feeling strength, which also enhanced readers’ attitudes. Finally, Experiment 1b offers two lines of evidence that the meanings inferred from swear words function as independent mediators: first, by conducting a factor analysis and, second, by showing that the indirect pathway for attribute strength can be turned off independently of feeling strength.

## Experiment 2: Swear Word Number

The purpose of Experiment 2 is to retest our full model (Figure 1) and to test the diagnosticity of attribute strength when manipulating swear word number (Figure 3). We expect that many (vs. few) swear words in a review should be less diagnostic of the product's attribute because it becomes unclear if the reviewer is swearing to convey the quantity of the product attribute or because of their disposition (e.g., they are prone to exaggerating). Thus, using a few swear words should lead to greater inferences about attribute strength than either many or no swear words. However, because swearing breaks a taboo (Stapleton 2010), using any swear words (few or many) should be similar in diagnosticity for the reviewer's feelings relative to no swear words. Overall, then, a review containing a few (vs. many vs. zero) swear words should have the greatest positive impact on readers’ product attitudes.

### Participants, Design, and Measures

We recruited 361 Prolific Academic participants, who completed the study in exchange for $.50 (M_age_ = 35.6 years; 49% male). We excluded 19 participants from analysis for failing an attention check (asking participants to report how the reviewer described the product's size), leaving a final sample of 342.

This study was a single-factor, three-level (number of swear words: zero vs. two vs. five) between-subjects design. We selected these conditions from the results of the swear word number variable in the field data. Specifically, we used two swear words because it had the strongest positive effect in the Yelp data, and because we wanted to extend our experimental findings (most other experiments use one swear word). We used the same scenario as Experiment 1a. In the zero swear words condition, the review title read, “It charged my phone fast.” In the two and five swear words conditions, the review title read, “Holy shit, it charged my phone fucking fast.” The remaining review text was the same in the zero and two swear words conditions: “But, it feels heavy. Still, it's handy and portable. It holds a charge fine and its size is okay.” In the five swear words condition, the text included three additional swear words that qualified other desirable product attributes: “But, it feels heavy. Still, the fucker is fucking handy and portable. It holds a charge damn fine and its size is okay.” After reading the review, participants answered questions related to product attitude (M = 5.57, SD = 1.03; α = .95), attribute strength (M = 5.58, SD = 1.17; α = .95) (charging speed), feeling strength (M = 5.54, SD = 1.40; α = .90), and offensiveness (Web Appendix G). All measures were as in Experiment 1a.

This study included some additional measures. First, the five swear words condition had three swear words that qualified other desirable product attributes: handiness, portability, and holding a charge. To test whether the swear words change the degree of these attributes, all participants reported on the degree to which the power station was handy (M = 5.75, SD = 1.19), portable (M = 5.55, SD = 1.28; 1 = “not at all,” and 7 = “very much”), and able to hold a charge (1 = “far below average,” and 7 = “far above average”; M = 5.36, SD = .98). Second, we predicted that five (vs. two) swear words in a review would be less diagnostic of attribute strength because it would be less clear if the reviewer used swear words to convey a quantity of the attribute or because of their disposition. Thus, we measured causal attribution using a scale adapted from He and Bond (2015). Participants in the two and five swear words conditions reported if they thought the swear words were caused by the reviewer's disposition toward swearing (1) or the reviewer's genuine assessment of the product (7). Higher (lower) scores indicated greater product (reviewer) attribution (M = 3.39, SD = 2.16).

### Results

A parallel mediation model with a multicategorical independent variable (Model 4; Hayes 2013) revealed significant effects when comparing two versus zero swear words and two versus five swear words.

#### Product attitude

An ANOVA revealed a significant total effect of swear word number on product attitude (F(2, 339) = 4.48, *p* = .012, $\eta_{p}^{2}$ = .026), such that participants liked the battery the most in the two swear words condition (M_two_ = 5.80, SD = .94), compared with both the zero (M_zero_ = 5.50, SD = .87; B = .38, SE = .13, t(339) = 2.20, *p* = .028) and five (M_five_ = 5.41, SD = 1.22; B = .30, SE = .13, t(339) = 2.84, *p* = .005) swear words conditions.

#### Attribute strength

An ANOVA on attribute strength (i.e., charging speed) also revealed a significant effect (F(2, 339) = 22.41, *p *< .001, $\eta_{p}^{2}$ = .117), such that participants perceived the battery to charge faster in the two swear words condition (M_two_ = 5.99, SD = 1.01) compared with the zero (M_zero_ = 5.04, SD = 1.19; B = .95, SE = .15, t(339) = 6.57, *p *< .001) and five (M_five_ = 5.69, SD = 1.10; B = .29, SE = .14, t(339) = 2.01, *p* = .045) swear words conditions.

ANOVAs on inferences about the other desirable attributes did not reveal significant effects for handy (F(2, 339) = 1.18, *p* = .31) or portable (F(2, 339) = 1.44, *p* = .24). The exception was ability to hold a charge (F(2, 338) = 3.84, *p* = .022, $\eta_{p}^{2}$ = .022), where participants perceived the battery to hold a charge better in the five swear words condition (M_five_ = 5.49, SD = .92) than the zero swear words condition (M_zero_ = 5.15, SD = .97; t(339) = 2.58, *p* = .01). However, as we expected, there was no difference between the five and two swear words conditions (M_two_ = 5.43, SD = 1.02; t(339) = .44, *p* = .66).

#### Feeling strength

An ANOVA on feeling strength was significant (F(2, 339) = 154.97, *p *< .001, $\eta_{p}^{2}$ = .478). Participants perceived the reviewer's feelings to be stronger in the two swear words condition (M_two_ = 6.18, SD = .82) than the zero swear words condition (M_zero_ = 4.16, SD = 1.30; B = 2.02, SE = .13, t(339) = 15.07, *p *< .001). As we expected, there was no difference in feeling strength between the two and five swear words conditions (M_five_ = 6.25, SD = .86; B = .07, SE = .13, t(339) = .51, *p* = .61).

#### Causal attribution

Only participants in the two and five swear words conditions reported on causal attribution. An ANOVA revealed a significant effect of swear word number on attribution (F(2, 227) = 6.90, *p* = .009, partial η^2^ = .030). Participants were more likely to attribute swearing to the reviewer than to the product when the review contained five swear words (M_five_ = 3.02, SD = 2.02) rather than two swear words (M_two_ = 3.72, SD = 2.24).

#### Mediation

The indirect effects of two (vs. zero) swear words via feeling strength (partial B = .50, SE = .11, 95% CI = [.28, .72]) and via attribute strength were significant (partial B = .37, SE = .07, 95% CI = [.24, .51]). Consistent with Experiments 1a and 1b, a pairwise comparison between the two indirect effects was not significant (partial B = .06, 95% CI = [−.31, .20]). The direct effect of swear words on product attitude remained significant when controlling for the mediators (B = −.60, SE = .14, 95% CI = [−.32, −.87]).

The indirect effect of two (vs. five) swear words via attribute strength was significant (i.e., charging speed; partial B = .11, SE = .06, 95% CI = [.01, .24]). As we expected, the indirect effect of two (vs. five) swear words via feeling strength was not significant (partial B = −.02, SE = .03, 95% CI = [−.07, .04]). The direct effect of swear words on product attitudes remained significant when controlling for the mediators (B = .28, SE = .11, 95% CI = [.07, .50]).

### Discussion

Experiment 2 tests the diagnosticity of attribute strength by comparing reviews containing zero, two, and five swear words. These results support our predictions, replicate the field data, and demonstrate process. Reviews with two (vs. zero) swear words increased inferences of the reviewer's feeling strength and the product's attribute strength (i.e., charging speed). These inferences independently and equally enhanced readers’ product attitudes. Reviews with two (vs. five) swear words conveyed the same feeling strength but greater attribute strength, which increased readers’ product attitudes. Further, while the five swear words condition contained swear words qualifying additional attributes (e.g., handy), there were no differences in inferences about these attributes across the swearing conditions. Together, these findings suggest that multiple swear words in a review reduce the diagnosticity of attribute strength. Indeed, participants attributed swearing to the reviewer when the review contained five (vs. two) swear words. Finally, this experiment offered new evidence that the two mediators function independently, by showing that the indirect pathway for inferences about the reviewer's feelings can be turned off independently of inferences about the product's attributes.

## Experiment 3: Swear Word Style

The purpose of Experiment 3 is to test the diagnosticity of attribute strength and feeling strength when manipulating swear word style (i.e., uncensored, censored, and euphemistic swear words; Figure 4). We expect a censored (vs. uncensored) swear word to be less diagnostic of attribute strength and feeling strength because it has a more positive connotation and does not break a taboo. It should therefore have a lower impact on readers’ products attitudes. However, there should be no difference between a euphemistic and an uncensored swear word.

This study retested arousal as an alternative explanation, as well as testing two new alternatives. First, we measured participant's arousal, this time using a multi-item measure (Mehrabian and Russell 1974). Second, because uncensored swear words are associated with truthfulness (Feldman et al. 2017; Hair and Ozcan 2018), they may increase product attitudes not because they convey meaning but because they make the reviewer more believable. Third, uncensored swear words can indicate shared group membership (Daly et al. 2004), which can affect purchase decisions (Berger and Heath 2008). Accordingly, if swear words increase readers’ perceptions of closeness with the reviewer, this could enhance product attitudes.

### Participants, Design, and Measures

We recruited 460 participants from MTurk using Cloud Research's vetted participant list (M_age_ = 41.3 years; 49% female). We excluded 25 participants for taking the survey more than once or for failing an attention check (asking participants to report how the reviewer described the product's size), leaving a final sample of 435.

This study was a single-factor, three-level (swear word style: uncensored [damn] vs. censored [d@mn] vs. euphemistic [darn]) between-subjects design. It used the same portable battery scenario as in Experiment 1. The review title used a degree adverb to manipulate swear word style: “It charged my phone [damn/d@mn/darn] fast.” The remaining text was the same across conditions: “But, it feels heavy. Still, it's handy and portable. It holds a charge fine and its size is okay.”

Participants then answered questions related to product attitude, attribute strength (charging speed), feeling strength, interpersonal closeness, arousal, believability, and offensiveness. Product attitude (M = 5.69, SD = .81; α = .94), attribute strength (M = 5.79, SD = 1.09; α = .96), feeling strength, (M = 5.05, SD = 1.18; α = .85) and offensiveness (Web Appendix G) were measured as Experiment 1a. Arousal was measured using four items with seven-point semantic differential scales, anchored as follows: “unaroused–aroused,” “sluggish–frenzied,” “calm–excited,” and “relaxed–stimulated” (Mehrabian and Russell 1974; M = 3.84, SD = 1.06; α = .81). Believability was measured using seven items adapted from prior research (e.g., “the reviewer is trustworthy”; 1–7 scales; Hair and Ozcan 2018; Lawrence, Fournier, and Brunel 2013; Poels, Janssens, and Herrewijn 2013; M = 5.68, SD = .90, α = .95). Interpersonal closeness between the participant and the reviewer was measured using Aron, Aron, and Smollan (1992) seven-point interpersonal closeness scale, wherein higher values indicate greater self-other overlap (M = 2.77; SD = 1.53).

### Results

A parallel mediation model with a multicategorical independent variable (Model 4; Hayes 2013) revealed significant effects when comparing an uncensored swear word with a censored swear word and an uncensored swear word with a euphemistic swear word.

#### Product attitude

An ANOVA revealed a significant total effect of swear word style on product attitude (F(2, 432) = 3.05, *p* = .049, $\eta_{p}^{2}$ = .014), such that participants liked the battery more in the uncensored swear word condition (M_uncensored_ = 5.82, SD = .76) compared with the censored swear word condition (M_censored_ = 5.58, SD = .84; B = .23, SE = .09; t(432) = 2.46, *p* = .014). As we expected, there was no difference between the uncensored and euphemistic swear word conditions (M_euphemistic_ = 5.68, SD = .80; B = .13, SE = .09; t(432) = 1.43, *p* = .15).

#### Attribute strength

An ANOVA on attribute strength (i.e., charging speed) revealed a significant effect (F(2, 432) = 3.63, *p* = .027, $\eta_{p}^{2}$ = .014), such that participants perceived the battery to charge faster in the uncensored swear word condition (M_uncensored_ = 5.98, SD = 1.00) compared with the censored swear word condition (M_censored_ = 5.66, SD = 1.13; B = .33, SE = .13; t(432) = 2.57, *p* = .011) and the euphemistic swear word condition (M_euphemistic_ = 5.73, SD = 1.13; B = .25, SE = .13; t(432) = 1.99, *p* = .048).

#### Feeling strength

Contrary to our expectations, an ANOVA on feeling strength was not significant (M_uncensored_ = 5.15, SD = 1.21; M_censored_ = 5.10, SD = 1.20; M_euphemistic_ = 4.91, SD = 1.14; F(2, 432) = 1.61, *p* = .20, partial η^2^ = .007).

#### Mediation

The indirect effect for an uncensored (vs. censored) swear word via attribute strength was significant (partial B = .12, SE = .05, 95% CI = [.03, .22]). The indirect effect via feeling strength was not (partial B = .01, SE = .03, 95% CI = [−.04, .07]). The direct effect was not significant when controlling for the mediators (B = .12, SE = .08, 95% CI = [−.03, .28]).

The indirect effect for an uncensored (vs. euphemistic) swear word via attribute strength was significant (partial B = .09, SE = .03, 95% CI = [.003, .19]). The indirect effect via feeling strength was not significant (partial B = .05, SE = .03, 95% CI = [−.04, .08]). The direct effect was not significant when controlling for the mediators (B = .02, SE = .08, 95% CI = [−.14, .18]).

#### Alternative explanations

Separate ANOVAs on arousal (M_uncensored_ = 3.99, SD = 1.07; M_censored_ = 3.77, SD = 1.02; M_euphemistic_ = 3.73, SD = 1.09; F(2, 432) = 2.60, *p* = .08, $\eta_{p}^{2}$ = .01), believability (M_uncensored_ = 5.70, SD = .84; M_censored_ = 5.64, SD = .94; M_euphemistic_ = 5.69, SD = .91; F(2, 432) = .20, *p* = .82, $\eta_{p}^{2}$ = .001), and interpersonal closeness were not significant (M_uncensored_ = 2.88, SD = 1.47; M_censored_ = 2.77, SD = 1.58; M_euphemistic_ = 2.66, SD = 1.55; F(2, 432) = .78, *p* = .46, $\eta_{p}^{2}$ = .003).

### Discussion

Experiment 3 tested the diagnosticity of attribute strength and feeling strength by manipulating swear word style. An uncensored (vs. censored) swear word led to inferences about the product's stronger attribute of charging speed, which independently enhanced readers’ attitudes. An uncensored (vs. euphemistic) swear word also led to inferences about the product's stronger charging speed, but this difference was not large enough to impact readers’ attitudes. Surprisingly, swear word style did not impact feeling strength. This finding suggests that any word that is close to breaking a taboo is sufficient to convey the reviewer's strong feelings.

## General Discussion

Although swear words have the potential to violate norms and to offend (e.g., Brown and Schau 2001; Dahl, Frankenberger, and Manchanda 2003; Hair and Ozcan 2018), this perspective does not fully capture their complexity. Further, the literal meanings of swear words have been lost over time, but it is not clear what swear words communicate beyond their original denotative meaning (Jay and Jay 2015), or how the meanings inferred from swear words might affect consumers. The current research fills this gap by using a linguistic lens to develop a nuanced model of how, why, and when swear words in reviews affect consumers. The data show that swear words change review helpfulness and readers’ attitudes toward the reviewed product. These results hold relative to reviews without swear words and reviews with non-swear-word synonyms. We find that inferences about the reviewer's feeling strength and the product's attribute strength function as independent, parallel, and equal mediators of this effect. Finally, we demonstrate that swear word number and style moderate these effects. We explored this model using Yelp and Amazon data, and in four experiments using various swear words, non-swear-word synonyms, and products (Table 5).

**Table 5.** Summary of Experimental Results.

Study	ANOVA Product Attitude	Comparison Conditions	Indirect Pathways	
			Reviewer Meaning	Product Meaning
1a	✓	Swear word (vs. no swear word)	✓	✓
1b	✓	Swear word (vs. non-swear-word synonym)	✓	✓
		Swear word (vs. negative word)	✓	✗
2	✓	Few (vs. no) swear words	✓	✓
		Few (vs. many) swear words	✗	✓
3	✓	Uncensored (vs. euphemistic) swear word	✗	✓
		Uncensored (vs. censored) swear word	✗^a^	✓

^a^ Does not support hypothesis.

*Notes:* ✓ = significant difference; ✗ = no difference.

The current work answers a call for research into factors that impact the reception of consumer reviews (Yelp 2017) by using a multimethod approach to demonstrate causal links between factors that reviewers and website moderators can control and consumer attitudes toward reviewed products. In doing so, we make several contributions to the literature.

First, we build on recent theoretical work suggesting that swear words as nouns may function as mixed-meaning expressions, conveying both speaker and topic meanings (e.g., “This asshole spilled their drink on me”; Löbner 2013). The current research compliments this theorizing by being the first to empirically test the meaning and impact of swear words. Specifically, we show that swear words convey these two meanings better than non-swear-word synonyms and can therefore play a pivotal role in product evaluations. Thus, this research identifies two new pathways via which swear words affect consumers and reveals that swear words are a particularly efficient communication tool.

Further, to our knowledge, this is the first research to empirically test these dual meanings in a single word. While prior work in linguistics has theorized about mixed-meaning expressions (i.e., words that convey meaning about the speaker and the topic; Gutzmann and Turgay 2012), it remains unclear how these dual meanings relate to one another. Likewise, while prior research in marketing (e.g., WOM, product design) shows that consumers make inferences about the speaker (e.g., Hamilton, Vohs, and McGill 2014) and the product (e.g., Kivetz and Simonson 2000) with implications for judgment and choice, much of this work has explored these inferences separately (Moore and Lafreniere 2020). Our work extends the linguistic and consumer behavior literatures by showing that a single word can simultaneously communicate information about different entities. The resulting inferences function as parallel mediators that can independently affect product attitudes. Perhaps even more critically, the current data show that inferences about the reviewer and the product affect reader's attitudes equally. This equivalence is somewhat surprising, given the relatively impoverished social context of reviews (Naylor, Lamberton, and Norton 2011), and suggests that marketing academics and practitioners should consider both product and reviewer inferences in WOM.

Third, the current work identifies two novel moderators that highlight when swearing is more helpful for readers. We suggest that the diagnosticity of swear words can influence readers of WOM, thereby distinguishing our interpretation of swear words from other more antisocial dynamics such as norm violations (Brown and Schau 2001; Dahl, Frankenberger, and Manchanda 2003) and offensiveness (Hair and Ozcan 2018). This diagnosticity perspective allows for distinct moderation predictions. Specifically, we examine the moderating role of swear word number (field data and Experiment 2) and swear word style (field data and Experiment 3). Our findings lead to an important implication both theoretically and practically: factors that change the meaning of swear words can mitigate their diagnosticity in reviews. This also has theoretical implications for mixed-meaning expressions: using a mixed-meaning expression is not enough—the expression also needs to offer discriminating information.

Fourth, the current research qualifies past work suggesting that swear words are only useful to readers when they are used in extremely positive reviews (Hair and Ozcan 2018). Indeed, review websites such as Amazon and Trip Advisor prohibit the use of swear words. The exception is Yelp, which allows swearing in reviews so long as the word(s) is not a threat, harassment, or hate speech. Consistent with Yelp’s policy, we show that, under certain conditions, swear words are useful to review readers regardless of review valence, but they are most effective in highly positive reviews. Specifically, a swear word in a negative review leads to negative inferences that increase review helpfulness and decrease readers’ attitudes toward the product. Similarly, a swear word in a positive review leads to positive inferences that increase review helpfulness and readers’ attitudes toward the product. The current research therefore suggests that website moderators may be wise not to ban swear words because they can increase the value of the review and readers’ attitude toward the reviewed product.

This work offers implications for platforms because it allows website moderators to predict when reviews with swear words may be more (or less) valuable. Specifically, while website moderators would do well to tolerate swear word use on their platform, they should not actively encourage swear word use. Instead, they could update their community guidelines to inform reviewers of the downside of using many swear words in their reviews. Further, website moderators may not benefit from removing or censoring swear words in reviews because censored and uncensored swear words are not interchangeable. Still, if platforms are concerned about offensiveness, censored swear words are sufficient to convey equally strong feelings as uncensored and euphemistic swear words. Our findings suggest that censorship still highlights the taboo topic, allowing readers to infer that the reviewer feels strongly. However, there is a trade-off because censorship do not convey comparable meaning about the product.

As this research is the first to empirically explore the meaning of swear words, there are several areas for future research. Foremost, the effects of censored swear words in reviews were not consistent. Experiment 3 showed a greater effect for uncensored swear words compared with censored swear words. Similarly, there was no effect for Amazon reviews when censored swear words were present versus absent. However, Yelp reviews received more helpful votes when censored swear words were present versus absent. This inconsistency could result from the different rules provided by Yelp and Amazon or from how readers attribute the censorship. Censored swear words may be more impactful if readers attribute the censorship to the website, rather than to the reviewer. Further, given that asterisks are often used to attract attention, censorship with many asterisks (e.g., holy s***) may be more effective than censorship with one asterisk (e.g., holy sh*t). More research is needed on the effect of censorship in reviews.

Future research should also consider the effect of swear words in different marketing contexts. For example, this model may not hold for reviews about products with inherently strong attributes or emotions (e.g., “Skydiving is damn fun!”) because the swear word's meanings may not offer diagnostic information. As another example, this model may not hold for swear words in advertising, because advertising content, compared with WOM, is typically exaggerated and emotionally intensified (Kronrod and Danziger 2013). Consequently, the meaning drawn from swear words may not be diagnostic in advertising, but other characteristics of swear words may be (e.g., shock; Dahl, Frankenberger, and Manchanda 2003).

Similarly, while we do not find support for alternative explanations such as arousal, believability, or attention, prior research has found support for these processes in other contexts (e.g., Feldman et al. 2017). It is possible that the lack of evidence is due to the form of measurement we used (e.g., attention could be measured via a scale) or due to the online review context, where swear words are prevalent and where readers make relatively cognitive judgments of reviewers (Moore and Lafreniere 2020). Researchers could continue to test these explanations as they explore the value of swear words in new areas, such as in face-to-face interactions.

While this research examines the diagnosticity of the swear word's meanings, other variables are likely to moderate the effect of swear words on product attitudes. For example, the frequency of swearing depends on demographic characteristics (Dewaele 2015). Thus, the effect of swear words (few or many) may be diminished if readers know that the reviewer is young. In this case, readers may attribute the swear word(s) to the reviewer's vernacular rather than the reviewer's feelings, thereby mitigating the effect of swear words on product attitudes.

Finally, our experiments test swear words in their most common form (i.e., degree adverbs). Swear words in other forms (e.g., nouns) should convey different meaning about the topic under discussion (e.g., Löbner 2013), which could alter the impact of swear words in reviews. Further, although we test our model using swear words directed at product attributes, person-directed swearing (i.e., slurs, pejoratives) are derogatory and may have different effects in reviews (e.g., “The people in charge here are truly the worst sons of bitches”; Hair and Ozcan 2018). Future research should test the effect of swear words in different grammatical forms and directed toward different entities. These questions and others await further investigation.

## Supplemental Material

sj-pdf-1-mrj-10.1177_00222437221078606 - Supplemental material for The Power of Profanity: The Meaning and Impact of Swear Words in Word of Mouth Supplemental material, sj-pdf-1-mrj-10.1177_00222437221078606 for The Power of Profanity: The Meaning and Impact of Swear Words in Word of Mouth by Katherine C. Lafreniere, Sarah G. Moore and Robert J. Fisher in Journal of Marketing Research
