# Supplementary material for: The Power of Profanity: The Meaning and Impact of Swear Words in Word of Mouth
Source: J Mark Res. 2022 Apr 28;59(5):908–25. doi: 10.1177/00222437221078606 (PMC13038166; doi:10.1177/00222437221078606)
Supplement: sj-pdf-1-mrj-10.1177_00222437221078606 - Supplemental material for The Power of Profanity: The Meaning and Impact of Swear Words in Word of Mouth [file sj-pdf-1-mrj-10.1177_00222437221078606.pdf]

WEB APPENDIX

The Power of Profanity: The Meaning and Impact of Swearwords in Word-of-Mouth

Katherine C. Lafreniere[1]

Sarah G. Moore[2]

Robert J. Fisher[3]

These materials have been supplied by the authors to aid in the understanding of their paper. The AMA is sharing these materials at the request of the authors.

[1] Katherine C. Lafreniere (lafreniere@uleth.ca) is Assistant Professor at the Dhillon School of Business, University of Lethbridge

[2] Sarah G. Moore (sarah.g.moore@ualberta.ca) is Professor of Marketing and Thornton A. Graham Chair at the Alberta School of Business, University of Alberta

[3] Robert J. Fisher (rfisher1@ualberta.ca) is Professor of Marketing and Chair of Marketing, Business Economics, and Law at the Alberta School of Business, University of Alberta

TABLE OF CONTENTS

A. Product categories in Amazon dataset ……………………………………………..…....3

B. Swearword dictionary used in field studies ……………………………….....…………..4

C. Yelp robustness checks ...………………………………………………...…………7

D. Amazon robustness checks ...…......……………………………….....……..………..11

E. Experiment manipulating review valence …………………...…………………..………....14

F. Stimuli of the swearword condition (Experiments 1A and 1B) ………………………..…..17

G. Manipulation checks on offensiveness (Experiments 1-3) ....………...…………………...18

H. Factor analysis: mediators and dependent variable 21

Web Appendix A: Product Categories in the Amazon Dataset

TABLE W1. PRODUCT CATEGORIES REPRESENTED IN THE AMAZON DATASET

**Product Category**	**Frequency**	**Percent**
Apps for Android	6,414	3.2
Automotive	3,301	1.7
Baby	2,360	1.2
Beauty	5,085	2.6
Books	56,088	28.0
CDs and Vinyl	9,367	4.7
Cell Phones and Accessories	8,467	4.2
Clothing, Shoes, and Jewelry	14,190	7.1
Digital Music	2,071	1.0
Electronics	19,499	9.7
Grocery and Gourmet Food	3,147	1.6
Health and Personal Care	7,309	3.7
Home and Kitchen	10,540	5.3
Instant Video	1,438	.7
Kindle Store	7,674	3.9
Movies and TV	11,550	5.8
Musical Instruments	1,188	.6
Office Products	3,090	1.5
Patio Lawn and Garden	2,344	1.2
Pet Supplies	3,099	1.5
Sports and Outdoors	8,218	4.1
Tools and Home Improvement	4,706	2.4
Toys and Games	5,549	2.8
Video Games	3,306	1.7
**Total**	**200,000**	**100%**

Web Appendix B: Swearword Dictionary used in Field Studies

The table of swearwords in the modified swearword dictionary is organized by uncensored, censored, and euphemistic swearwords (Table W2). A diamond (◆) denotes the acceptance of all letters, hyphens or numbers following its appearance. For example, the dictionary includes the word shit◆, which allows for any word that matches the first four letters to be counted as a swearword (including shithead, shitty, shits). To ensure accuracy, we had to manually check the results of ambiguous word stems using Microsoft Excel's search function (e.g., *d**** in reviews could be used to censor the swearword *dick* or to call attention to a passage). All censored swearwords represented by symbols (e.g., "It was f@#$%^ amazing!") had to be manually added using Excel's search function because the number and order of symbols were not consistent and the symbols did not always reflect a swearword (e.g., symbols contained in a website address). We found these censored swearwords by searching for all combinations of words containing at least two of the following symbols: !@#$%^&* (e.g., "s#!t"). Further, swearwords that could be categorized as either euphemistic or censored were categorized as euphemistic if they were pronounceable (e.g., "a-hole"). The phrase of an acronym is in brackets.

TABLE W2. MODIFIED SWEARWORD DICTIONARY

Uncensored	*Euphemistic*	*Censored*
assh◆	a-hole	@$$◆
assl◆	arse◆	@hole
assp◆	biatch◆	@ss
asses	biotch◆	a$$
ass	cox	a**◆
assf◆	d-bags	bad@ss
bastard	dang◆	bullsh*t◆
bullshit◆	darn	bull$#!t

bitch◆	effin◆	b*tch◆
bombass	eff	b**ch◆
boob◆	fock	b***h◆
butt	faak	b****
butts	fakkin◆	b**b◆
clusterfuck	fark	bs (bullshit)
cock◆	fawk	c*ck
crap	fcuk	c*nt◆
crappy	freak-en	c**t◆
cunt◆	freakin◆	c***
dam	freggin◆	clusterf◆
damn◆	frig◆	d@mn
dammit	frick◆	d*mn◆
dick◆	frikkin	d*ck◆
douche◆	fugg◆	d**k◆
dumbass	fuk◆	d***
dumbf◆	heck	f off (fuck off)
fuck◆	holy moly	F’ing
goddam◆	Mofo (mother fucker)	f---
hell	pho’ck’d	f—k
hella	shiznit	f*ck◆
kickass	sux	f**k◆
kissass		f***◆
jackass◆		F word
jerk		f-you
lameass◆		FFS (for fuck sakes)
motherf◆		Fkn
mothaf◆		fml (fuck my life)
nigger◆		Gtfo (get the fuck out)
piss◆		h*ll◆
prick◆		h***
pussy◆		Idgaf (I don’t give a fuck)
sonofa◆		Idgad (I don’t give a damn)
suck◆		Lmao (laugh my ass off)
shit◆		Lmfao (laugh my fucking ass off)
tit		n*gger◆
tits		Omfg (oh my fucking God)
titties		p*ssy◆
titty		p***y◆
twat		p****
wanker◆		$#!t
		$hiznit
		s---
		sh*t◆
		s**t
		s***

		sh**◆<br>sh!t<br>Smfh (shake my fucking head)<br>Stfu (shut the fuck up)<br>wtf (what the fuck)<br>wth (what the hell)<br><br>Example Combinations:<br>@#<br>&^<br>@^<br>$&<br>$@<br>F&<br>F#<br>f@<br>B#

Web Appendix C: Yelp Robustness Checks

We conducted a robustness check to see if the results of the Yelp field data would hold if the star ratings variable was modelled as a categorical variable (3 stars = 0, 1 or 2 stars =1, 4 or 5 stars = 2). Consistent results for the Yelp data were obtained when star ratings were modeled as a three-level categorical variable (B = .320, Wald X^2 (1, n = 100,000) = 319.95, $p < .001$; Table W3).

TABLE W3. USEFUL VOTES AS A FUNCTION OF SWEARWORDS IN YELP REVIEWS

Variables	*Coefficient*	*(SE)*
Swearwords	.320***	(.018)
Controls		
Months Posted	.007***	(.0002)
Review Length[1]	.00001***	(.0000001)
Negative Review	.186***	(.019)
Positive Review	-.062***	(.017)
Dispersion	1.76*	(.016)
Pearson X^2	193,265.91	

[1] Square transformed, *$p < 0.05$, **$p < .01$, ***$p < .001$, N = 100,000

We also checked if the results of the Yelp field data would hold if swearwords were identified using the original (i.e., unmodified) swearword dictionary from LIWC (Tausczik and Pennebaker 2010). The dependent variable (number of useful votes) and the control variables (review length, review valence, and months posted) remained the same. Of the 100,000 randomly selected reviews, the unmodified dictionary identified 7,771 (7.7%) reviews containing at least one swearword (the modified dictionary identified 8,348 reviews containing at least one swearword). Descriptive statistics using the unmodified dictionary are presented in Table W4.

TABLE W4. YELP DESCRIPTIVE STATISTICS (UNMODIFIED DICTIONARY)

Variable	*Total* *Mean (SD)*	*Swearwords Present* *Mean (SD)*	*Swearwords Absent* *Mean (SD)*
Useful Votes	1.01 (2.44)	1.87 (4.14)	.94 (2.22)
Review Length	117.37 (109.53)	202.17 (159.24)	110.23 (101.08)
Months Posted	32.01 (25.66)	39.34 (28.65)	31.39 (25.29)
Review Valence	3.73 (1.40)	3.02 (1.56)	3.79 (1.37)
	Total *Count*	*Swearwords Present* *Count*	*Swearwords Absent* *Count*
N	100,000	7,771	92,229
Negative Reviews	21,583	3,172	18,411
Neutral Reviews	12,448	1,035	11,413
Positive Reviews	65,969	3,564	62,405

We modeled the value of the review using negative binomial regression ($M = 1.01$, Var = 5.94, dispersion coefficient = 1.76, CI 1.73 to 1.79). Controlling for review length, months posted, and review valence, we found similar results to those using the modified swearword dictionary. Specifically, reviews containing swearwords received more useful votes than reviews without swearwords ($B = .332$, Wald X^2 (1, n = 100,000) = 308.09, $p < .001$). Consistent results were obtained when swearwords were modeled as a continuous variable using the number of swearwords as a proportion of review length ($B = .192$, Wald X^2 (1, n = 100,000) = 233.16, $p < .001$), when star ratings were modeled as a categorical variable ($B = .335$, Wald X^2 (1, n = 100,000) = 311.66, $p < .001$), and when the dependent variable was the percentage of swearwords in a review (Yelp: B = .06, SE = .01, Wald X^2 (1, n = 100,000) = 20.33, $p < .001$). The results are summarized in Table W5.

TABLE W5. PRESENCE OF SWEARWORDS ON YELP USEFUL VOTES (UNMODIFIED DICTIONARY)

	Discrete Model				Continuous Model	
	Star Ratings (Continuous)		Star Ratings (Categorical)		Star Ratings (Continuous)	
Variables	*Coefficient*	*(SE)*	*Coefficient*	*(SE)*	*Coefficient*	*(SE)*
Swearwords	.332***	(.019)	.335***	(.019)	.192***	(.013)
Controls						
Review Length[1]	.00001***	(.0000001)	.00001***	(.0000001)	.00001***	(.0000001)
Months Posted	.007***	(.0002)	.007***	(.0002)	.007***	(.0002)
Review Valence	-.080***	(.004)			-.080***	(.004)
Negative Review			.181***	(.019)		
Positive Review			-.060***	(.016)		
Dispersion	1.75*	(.016)	1.76*	(.016)	1.76*	(.016)
Pearson X^2	192467.38		192324.30		189760.82	

[1] Square transformed, $^{*}p < 0.05$, $^{**}p < .01$, $^{***}p < .001$, N = 100,000

We checked if the results of the Yelp field data would hold if the same swearword were used multiple times in a review. We coded the use of individual swearwords as a categorical variable (e.g., 0 = no use of the word *fuck*; 1 = one use, 2 = two uses, etc.). Controlling for months posted, review valence, and review length, a review containing one (vs. zero) use of the word *fuck* had a positive effect on useful votes (B = .88, SE = .11, Wald X^2 (1, n = 100,000) = 65.41, $p < .001$, n = 178). A review containing two uses (vs. zero) of the word *fuck* also had a significant and positive effect: B = .54, SE = .27, Wald X^2 (1, n = 100,000) = 3.91, $p = .04$, n = 30. A review containing three uses of the word *fuck* had no significant effect (B = .35, SE = .06, Wald X^2 (1, n = 100,000) = .35, $p = .55$, n = 6). There were only three reviews containing four uses of the word *fuck*. Similar results were found when the individual swearword was *damn* and *shit*. It seems that the number of swearwords in a review has the same curvilinear effect

regardless of word repetition. We note that swearword repetition may exacerbate the effect, but suspect it would not change its direction or the mediation results.

Finally, we checked if the results of the Yelp field data would hold if the comparison condition were non-swearwords. The challenge here is to identify appropriate comparisons. We tackled this in two ways. First, we tested some of the comparison words from our experiments. For example, we recategorized the presence vs. absence of the word *fuck* and *extremely* into one categorical variable (0 = presence of extremely, 1 = neither present, 2 = presence of fuck, 3 = presence of both). Yelp reviews containing the word *fuck* (vs. *extremely*) received significantly more useful votes (B = .67, SE = .11, Wald X^2 (1, 100,000) = 40.86, $p < .001$). Similar results were found when the control condition was the negative word *insanely*: B = .39, SE = .16, Wald X^2 = 1, 100,000) = 6.20, $p = .01$. We did not test *super* because it is much more likely than the others to be used as a non-degree adverb (e.g., Supersize, Super Bowl, Super Mario). Second, we compiled a list of 48 common degree adverbs from a linguistic website and created an all-inclusive degree adverb variable that we could add to our original continuous model. We found that the standardized B coefficient for the all-inclusive swearword variable (exponentiated B = 1.18; B = .17, SE = .01, Wald X^2 (1, n = 100,000) = 207.11, $p < .001$) was larger than the standardized B coefficient for the all-inclusive degree adverb variable (exponentiated B = 1.07, B = .06, SE = .002, Wald X^2 (1, n = 100,000) = 840.83, $p < .001$).

While we hope these analyses offer some insight, we have not included them in the paper due to space limitations. More importantly, we do not report them because these results are not causal, and they do not offer a clean test: we cannot be sure that all instances of the swearword or the comparison word were degree adverbs. Fortunately, our experiments overcome these limitations, offering a clean and precise test of the basic effect and its underlying process.

Web Appendix D: Amazon Robustness Checks

We conducted a robustness check to see if the results of the Amazon field data would hold if the star ratings variable was modelled as a categorical variable (3 stars = 0, 1 or 2 stars =1, 4 or 5 stars = 2. Consistent results for the Amazon data were obtained when star ratings were modeled as a three-level categorical variable (*B* = .150, Wald X^2 (1, n = 200,000) = 14.98, $p < .001$; Table W6).

TABLE W6. PROPORTION OF HELPFUL VOTES AS A FUNCTION OF SWEARWORDS IN AMAZON REVIEWS

Variables	*Coefficient*	*(SE)*
Swearwords	.150***	(.04)
Controls		
Months Posted	.011***	(.0002)
Review Length[1]	.000001***	(.0000001)
Negative Review	.189***	(.03)
Positive Review	.05*	(.03)
Dispersion	9.83*	(.041)
Pearson X^2	44,787.58	

[1] Square transformed, *$p < 0.05$, **$p < .01$, ***$p < .001$, N = 200,000

We conducted another robustness check to see if the results of the Amazon field data would hold if swearwords were identified using the original (i.e., unmodified) swearword dictionary from LIWC (Tausczik and Pennebaker 2010). The dependent variable (proportion of helpful votes) and the control variables (review length, review valence, and months posted) remained the same. Of the 200,000 randomly selected reviews, the unmodified dictionary identified 8,805 (4.4%) reviews containing at least one swearword (the modified dictionary identified 6,608 reviews containing at least one swearword). Descriptive statistics using the unmodified dictionary are presented in Table W7.

TABLE W7. AMAZON DESCRIPTIVE STATISTICS (UNMODIFIED DICTIONARY)

Variable	*Total* *Mean (SD)*	*Swearwords Present* *Mean (SD)*	*Swearwords Absent* *Mean (SD)*
Proportion of Helpful Votes	32.82 (43.58)	45.26 (43.83)	32.24 (43.49)
Review Length	91.94 (123.85)	224.41 (257.83)	85.84 (110.17)
Months Posted	28.59 (35.56)	38.44 (41.58)	28.13 (35.19)
Review Valence	4.17 (1.25)	3.70 (1.51)	4.19 (1.24)
	Total *Count*	*Swearwords Present* *Count*	*Swearwords Absent* *Count*
N	200,000	8,805	191,195
Negative Reviews	26,053	2,159	23,894
Neutral Reviews	17,154	882	16,272
Positive Reviews	156,793	5,764	151,029

We modeled the value of the review using negative binomial regression (M = 32.82, Var = 1,899.82, dispersion coefficient = 9.83, CI: 9.75 to 9.91). Controlling for review length, months posted, and review valence, we found similar results to those using the modified swearword dictionary. Specifically, reviews containing swearwords received a higher proportion of helpful votes than reviews without swearwords (B = .180, Wald X^2 (1, n = 200,000) = 26.80, p < .001). Consistent results were obtained when swearwords were modeled as a continuous variable using the number of swearwords (B = .097, Wald X^2 (1, n = 200,000) = 17.98, p < .001) and when star ratings were modeled as a categorical variable (B = .176, Wald X^2 (1, n = 200,000) = 25.65, p < .001). The results are summarized in Table W8.

TABLE W8. PRESENCE OF SWEARWORDS ON AMAZON PROPORTION OF HELPFUL VOTES (UNMODIFIED DICTIONARY)

	Discrete Model				Continuous Model	
	Star Ratings (Continuous)		Star Ratings (Categorical)		Star Ratings (Continuous)	
Variables	*Coefficient*	*(SE)*	*Coefficient*	*(SE)*	*Coefficient*	*(SE)*
Swearwords	.180***	(.035)	.097***	(.023)	.097***	(.012)
Controls						
Review Length[1]	.000001***	(.0000001)	.000001***	(.0000001)	.000001***	(.0000001)
Months Posted	.011***	(.0002)	.011***	(.0002)	.011***	(.0002)
Review Valence	-.024***	(.006)			-.025***	(.006)
Negative Review			.188***	(.031)		
Positive Review			.051*	(.025)		
Dispersion	9.83*	(.041)	9.83*	(.041)	9.83*	(.041)
Pearson X^2	44683.27		44830.04		44645.19	

[1]Square transformed, *$p < 0.05$, **$p < .01$, ***$p < .001$, N = 200,000

Web Appendix E: Experiment Manipulating Review Valence

The purpose of this study was to retest the notion that swearwords in reviews are valuable to readers regardless of review valence. We hypothesized that a swearword qualifying a desirable product attribute would have a positive effect on readers' attitudes towards the reviewed product, while a swearword qualifying an undesirable product attribute would have a negative effect.

This study was a 2 (swearword: present vs. absent) by 2 (review valence: negative vs. positive) between-subjects design. Three hundred and ninety-eight individuals from Amazon's Mechanical Turk (MTurk; M_{age} = 36.9; 48% male) were recruited to participate. Thirty-four were excluded from analysis for failing the attention check (participants were asked to report how the reviewer described the product's size), leaving a final sample of 364.

Participants were asked to imagine that they wanted to buy a new external battery (i.e., power station) for their electronic devices. Then they were shown a seller's website with an image and a product description of an external battery, along with one review. In the positive review condition, the product was rated 4 out of 5 stars. The title read, "It charged my phone fucking fast," and the text of the review read, "It's handy and portable. But it feels heavy. It holds a charge fine and its size is okay." In the negative review condition, the product was rated 2 out of 5 stars. The title read, "It charged my phone fucking slow," and the text of the review read, "It's not handy or portable. But it feels light. It holds a charge fine and its size is okay." The swearword was omitted from the title in the swearword absent condition.

As a manipulation check, participants were asked if they found the review to be offensive (1 = *not at all*, 7= *very much*; *M* = 2.03, SD = 1.65) and if they thought most people would find the review to be offensive (1 = *not at all*, 7= *very much*; *M* = 2.69, SD = 1.96). Participants'

attitude towards the power station was measured with six items using seven-point semantic differential scales with the following anchors: negative—positive, dislike—like, good—bad, unfavorable—favorable, unappealing—appealing, and unpleasant—pleasant (M = 4.49, SD = 1.55, α = 0.98). Participants also reported on a sliding scale how much (in dollars) they would pay for the power station on a \$0 to \$200 scale (M = \$34.10, SD = \$27.70).

Results

Offensiveness. A full factorial ANOVA with review offensiveness as the dependent variable showed a significant main effect of swearword (M_{fucking} = 2.77, SD = 1.90; M_{control} = 1.32, SD = .93; $F(1, 361) = 86.10$, $p < .001$, partial $\eta^2 = .193$). Neither the main effect of review valence ($F(1, 361) = 1.53$, $p = .22$) nor the interaction ($F(1, 361) = .006$, $p = .94$) were significant. Further, a full factorial ANOVA with perceived offensiveness of the review to others as the dependent variable showed significant effects of swearword (M_{fucking} = 3.98, SD = 1.76; M_{control} = 1.44, SD = 1.18; $F(1, 360) = 262.59$, $p < .001$, partial $\eta^2 = .422$) and valence (M_{negative} = 2.88, SD = 2.03; M_{positive} = 2.50, SD = 1.88; $F(1, 360) = 5.32$, $p = .022$, partial $\eta^2 = .015$). The interaction was not significant ($F(1, 360) = .0002$, $p = .99$).

Product attitudes. A full factorial ANOVA showed a significant main effect of review valence on product attitudes ($F(1, 361) = 410.16$, $p < .001$, partial $\eta^2 = .532$) and a significant interaction ($F(1, 361) = 8.88$, $p = .003$, partial $\eta^2 = .024$). When the review was positive, participants held more favorable product attitudes when the swearword was present (M_{fucking} = 5.80, SD = .91) versus absent (M_{control} = 5.45, SD = .86, $t(361) = 2.22$, $p = .027$, partial $\eta^2 = .014$). When the review was negative, participants held less favorable product attitudes when the swearword was present (M_{fucking} = 3.22, SD = 1.05) versus absent (M_{control} = 3.53, SD = 1.35,

t(361) = 1.99, p = .048, partial η^2 = .011). These results hold when controlling for offensiveness, F(1, 358) = 8.68, p = .003, partial η^2 = .024.

Willingness-to-pay. An ANOVA on willingness-to-pay revealed a significant main effect of review valence (F(1, 361) = 44.43 p < .001, partial η^2 = .110) as well as a significant interaction (F(1, 361) = 8.32 p = .004, partial η^2 = .023). When the review was positive, participants were willing to pay more for the product when the swearword was present (M_{fucking}= \$47.34, SD = \$29.46) versus absent (M_{control} = \$39.18, SD = \$25.50, t(361) = 2.11, p = .036, partial η^2 = .012). When the review was negative, participants were willing to pay less for the product when the swearword was present (M_{fucking}= \$21.34, SD = \$16.65) versus absent (M_{control} = \$28.89, SD = \$30.14, t(361) = 1.97, p = .05, partial η^2 = .011). These results hold when controlling for offensiveness, F(1, 358) = 7.95, p = .005, partial η^2 = .022.

Discussion

The results of this study provided support for our theorizing that swearwords are valuable to review readers. The presence (vs. absence) of a swearword affected readers' attitudes towards—and willingness-to-pay for—the product under review. Specifically, in a positive review where the swearword qualified a desirable product attribute, readers held more favorable product attitudes and were willing to pay more for the reviewed product. However, in a negative review where the swearword qualified an undesirable product attribute, readers held less favorable product attitudes and were willing to pay less for the reviewed product. In short, this study demonstrated that relative to no swearwords, swearwords can complement both positive or negative effects on review readers.

Web Appendix F: Stimuli of the Swearword Condition (Experiments 1A and 1B)

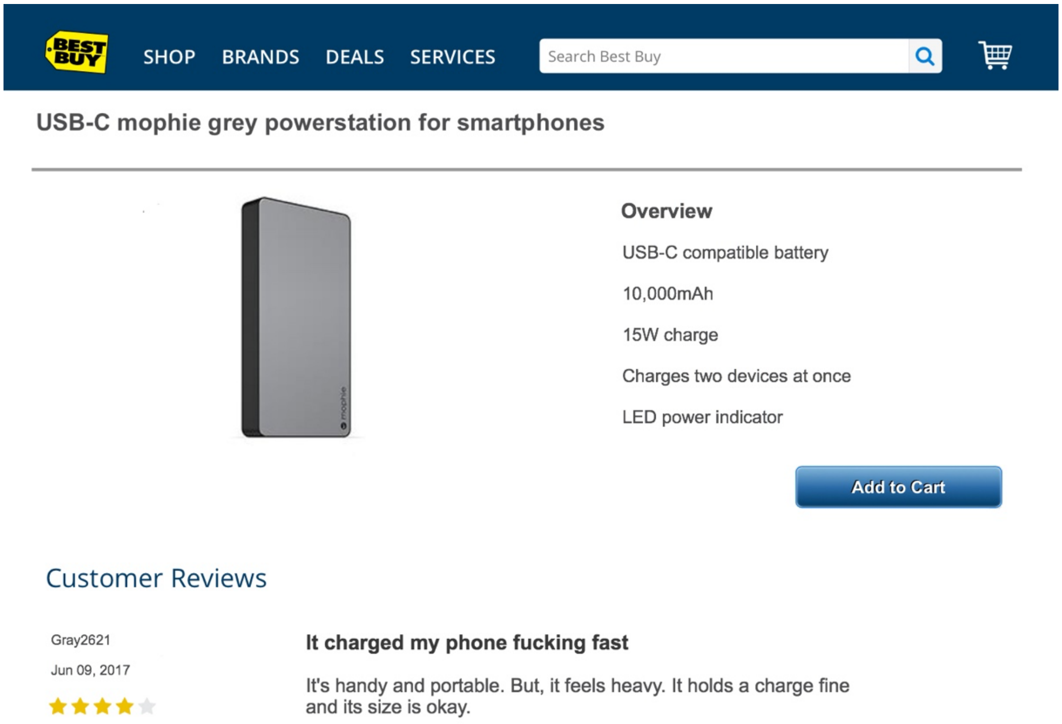

Figure W1: Experiment 1A

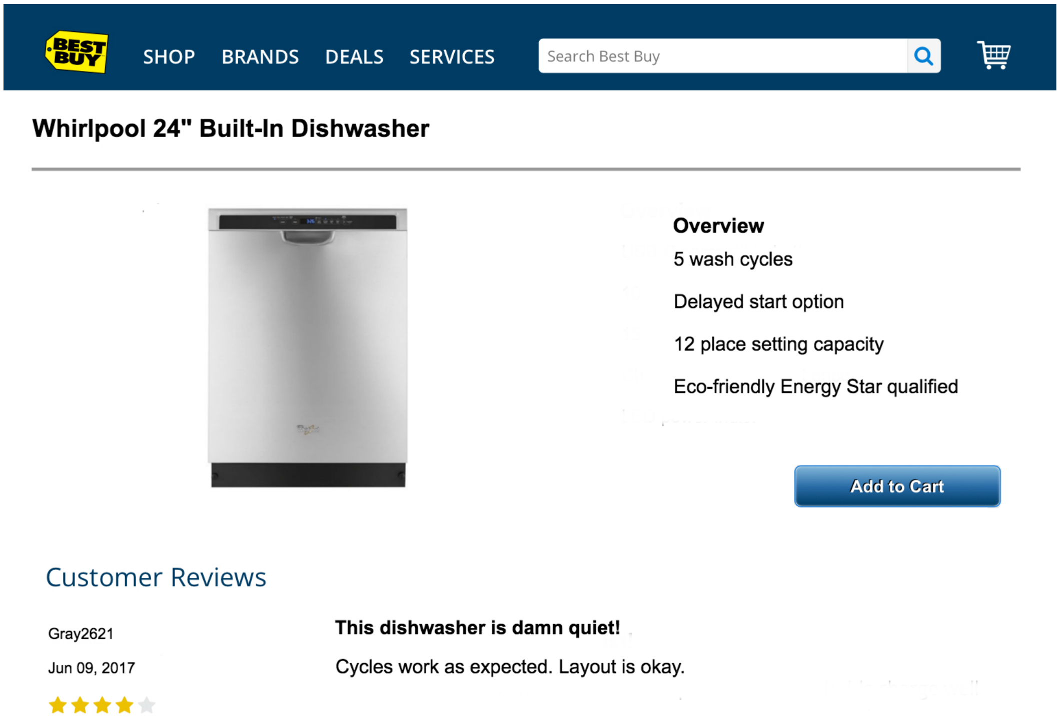

Figure W2: Experiment 1B

Web Appendix G: Manipulation Checks on Offensiveness (Experiments 1-3)

Experiment 1A (swearword vs. no swearword). One-way ANOVAs on review offensiveness ($M_{fucking}$ = 3.18, SD = 2.29, $M_{control}$ = 1.21, SD = .69; $F(1, 183) = 62.49$, $p < .001$, partial $\eta^2 = .255$) and perceived offensiveness of the review to others ($M_{fucking}$ = 4.26, SD = 1.79, $M_{control}$ = 1.29, SD = .67; $F(1, 183) = 222.12$, $p < .001$, partial $\eta^2 = .548$) both revealed a significant effect of swearword; participants found the review more offensive and perceived others to be more offended when the swearword was present versus absent. The results for product attitudes hold when controlling for offensiveness, $F(1, 182) = 9.50$, $p = .002$, partial $\eta^2 = .050$.

Experiment 1B (swearword vs. non-swearword synonyms). Manipulation checks were measured using offensiveness (review offensiveness [M = 1.53, SD = 1.21] and perceived review offensiveness to most people [M = 1.50, SD = 1.07]). A one-way ANOVA revealed a significant effect on review offensiveness ($F(2, 288) = 3.61$, $p = .028$, partial $\eta^2 = .024$). The review was more offensive in the swearword condition (M_{damn} = 1.78, SD = 1.46) compared to the mixed-meaning word condition (M_{super} = 1.32, SD = .87, $t(288) = 2.65$, $p = .009$) and the negative word condition ($M_{insanely}$ = 1.48, SD = 1.18), $t(288) = 1.71$, $p = .087$). For perceived offensiveness to others, an ANOVA also revealed a significant effect ($F(2, 288) = 24.36$, $p < .001$, partial $\eta^2 = .145$), such that participants perceived others to be more offended in the swearword condition (M_{damn} = 2.56, SD = 1.61), relative to the mixed-meaning word (M_{super} = 1.39, SD = 1.02, $t(288) = 6.39$, $p < .001$) and the negative word conditions ($M_{insanely}$ = 1.53, SD = 1.15), $t(288) = 5.63$, $p < .001$). The results for product attitude hold when controlling for offensiveness ($F(1, 287) = 10.73$, $p < .001$, partial $\eta^2 = .070$).

Experiment 2 (swearword number). Offensiveness (review offensiveness [$M = 1.53$, SD = 1.21] and offensiveness to most people [$M = 3.54$, SD = 2.11]) was measured as in study 2. A one-way ANOVA revealed a significant effect for the number of swearwords on review offensiveness ($F(2, 339) = 23.97$, $p < .001$, partial $\eta^2 = .124$): the review was more offensive in the two swearwords condition ($M_{two} = 2.70$, SD = 2.01) compared to the zero swearwords condition ($M_{zero} = 1.42$, SD = 1.20; $t(339) = 5.46$, $p < .001$). There was no difference between the two and five swearwords conditions ($M_{five} = 2.93$, SD = 1.94; $t(339) = .99$, $p = .32$). For perceived offensiveness of the review to most people, an ANOVA also revealed a significant effect ($F(2, 339) = 169.49$, $p < .001$, partial $\eta^2 = .500$). Participants perceived most others to be more offended in the two ($M_{two} = 4.15$, SD = 1.78) versus the zero swearwords condition ($M_{zero} = 1.47$, SD = 1.20, $t(339) = 13.53$, $p < .001$), but more offended in the five versus the two swearwords condition ($M_{five} = 4.97$, SD = 2.11; $t(339) = 4.18$, $p < .001$). The results for product attitude hold when controlling for offensiveness ($F(2, 338) = 4.47$, $p = .012$, partial $\eta^2 = .026$).

Experiment 3 (uncensored, euphemistic, and censored swearwords). Manipulation checks were measured using review offensiveness (i.e., “Do you find this review to be offensive” on a 1 [not at all] to 7 [very much] scale; $M = 1.26$, SD = 0.70) and perceived offensiveness to most people (i.e., “Do you think most people would find this review to be offensive,” on a 1 (not at all) to 7 (very much) scale; $M = 1.54$, SD = 1.02). A one-way ANOVA revealed a significant effect for the swearword style on review offensiveness ($F(2, 431) = 21.88$, $p < .001$, partial η^2 = .09): the review was more offensive in the uncensored swearword condition ($M_{uncensored} = 1.29$, SD = .75) compared to the euphemistic swearword condition ($M_{euphemistic} = 1.07$, SD = .70; $t(431) = 2.71$, $p = .008$). There was no difference between the uncensored and censored swearword conditions ($M_{censored} = 1.42$, SD = .85; $t(431) = 1.60$, $p = .11$). For perceived offensiveness of the

review to most people, an ANOVA also revealed a significant effect ($F(2, 431) = 21.88$, $p < .001$, partial $\eta^2 = .092$). Participants perceived most others to be more offended in the uncensored swearword ($M_{uncensored} = 1.61$, SD = 1.04) versus the euphemistic swearwords condition ($M_{euphemistic} = 1.13$, SD = .63, $t(431) = 4.22$, $p < .001$), but less offended in the uncensored versus censored swearword condition ($M_{censored} = 1.88$, SD = 1.17; $t(431) = 2.33$, $p = .02$). The results for product attitude hold when controlling for offensiveness ($F(2, 430) = 2.90$, $p = .05$, partial $\eta^2 = .013$) and perceived offensiveness of the review to most people ($F(2, 430) = 2.88$, $p = .05$, partial $\eta^2 = .013$).

Web Appendix H: Factor Analysis: Mediators and Dependent Variable

Experiment 1A (swearword vs. no swearword). A principal-component analysis with varimax rotation[1] was conducted on the items comprising the two inferred meaning measures and the product attitude measure; three factors emerged. The first factor captured product attribute intensity (31.85% of variance, eigenvalue = 7.33). All 6 items assessing product attribute intensity (i.e., charging speed) had a factor loading above .81 on the same factor and a factor loading below .22 on the other factors. The second factor captured product attitudes (29.35% of variance, eigenvalue = 2.60). All 6 items assessing product attitudes had a factor loading above .75 on the same factor and a factor loading below .20 on the other factors. The third factor captured reviewer feeling strength (15.94% of variance, eigenvalue = 1.49). All 3 items assessing reviewer feeling strength had a factor loading above .75 on the same factor and a factor loading below .26 on the other factors.

Experiment 1B (swearword vs. non-swearword synonym). A principal-component factor analysis with varimax rotation was conducted on the inferred meanings and product attitude items; three factors emerged. The first factor captured product attitude (29.3% of variance, eigenvalue = 6.53). All 6 items for product attitude loaded above .83 on this factor and below .17 on the others. The second factor captured was attribute strength (i.e., quietness; 28.7% of variance, eigenvalue = 3.46). All 7 items for attribute strength loaded above .72 on this factor and below .23 on the others. The third factor captured feeling strength (15.9% of variance, eigenvalue = 1.83). All 3 items for feeling strength loaded above .81 on this factor and below .21 on the others.

---

[1] A factor analysis with oblimin rotation produced similar results across studies.

Experiment 2 (swearword number). A principal-component analysis with varimax rotation was conducted on the items composing the two inferred meanings measures and the product attitude measure; three factors emerged. The first factor captured product attitude (32.49% of variance, eigenvalue = 7.85). All 6 items comprised of product attitude had a factor loading above .82 on the same factor and a factor loading below .24 on the other factors (eigenvalue = 7.85). The second factor captured attribute strength (i.e., charging speed; 31.92% of variance, eigenvalue = 2.72). All 6 items comprised of attribute strength (i.e., charging speed) had a factor loading above .81 on the same factor and a factor loading below .24 on the other factors. The third factor captured was feeling strength (17.23% of variance, eigenvalue = 1.68) All 3 items comprised of feeling strength had a factor loading above .79 on the same factor and a factor loading below .23 on the other factors.

Experiment 3 (uncensored, euphemistic, and censored swearwords). A principal-component analysis with varimax rotation was conducted on the items composing the two inferred meanings measures and the product attitude measure; three factors emerged. The first factor captured attribute strength (i.e., charging speed; 32.82% of variance, eigenvalue = 7.92). All 6 items comprised of attribute strength had a factor loading above .82 on the same factor and a factor loading below .27 on the other factors (eigenvalue = 7.85). The second factor captured product attitude (30.75% of variance, eigenvalue = 2.37). All 6 items comprised of product attitude had a factor loading above .80 on the same factor and a factor loading below .29 on the other factors. The third factor captured was feeling strength (16.16% of variance, eigenvalue = 1.68) All 3 items comprised of feeling strength had a factor loading above .76 on the same factor and a factor loading below .26 on the other factors.
